# Extensive intraoperative peritoneal lavage (EIPL) for gastric cancer with positive peritoneal lavage and/or stamp cytology: An exploratory phase II study

**DOI:** 10.1371/journal.pone.0347742

**Published:** 2026-04-17

**Authors:** Gen Tsujio, Masakazu Yashiro, Yuichiro Miki, Kohei Matsuoka, Koji Maruo, Mami Yoshii, Tatsuro Tamura, Katsunobu Sakurai, Takahiro Toyokawa, Naoshi Kubo, Shigeru Lee, Tomohisa Okuno, Kishu Kitayama, Go Masuda, Masaichi Ohira, Kiyoshi Maeda

**Affiliations:** 1 Department of Gastroenterological Surgery, Osaka Metropolitan University Graduate School of Medicine, Osaka, Japan; 2 Molecular Oncology and Therapeutics, Osaka Metropolitan University Graduate School of Medicine, Osaka, Japan; 3 Cancer Center for Translational Research, Osaka Metropolitan University Graduate School of Medicine, Osaka, Japan; 4 Department of Gastroenterological Surgery, Osaka City General Hospital, Osaka, Japan; Athens Medical Group, Psychiko Clinic, GREECE

## Abstract

**Background:**

Our group revealed that the combination of intra-operative stamp cytology and peritoneal lavage cytology (CY) improved the identification of individuals with high risk of peritoneal metastasis. In this exploratory Phase II study, we aimed to evaluate the effect on relapse-free survival (RFS) of extensive intraoperative peritoneal lavage (EIPL) for gastric cancer with positive peritoneal cytology (CY1) and/or stamp cytology positive (stamp+).

**Materials and methods:**

This study was a single arm, multi-institutional, exploratory phase 2 trial to assess the effects of EIPL after open gastrectomy for gastric cancer with CY1 and/ or stamp+. The primary endpoint was RFS. Secondary endpoints were overall survival (OS), postoperative recurrence site and incidence of postoperative adverse events.

**Results:**

Between 2017 and 2021, 13 patients from 2 institutions were enrolled in this study. Because of the recent decline in open abdominal surgery, the number of cases did not increase and the trial was closed due to lack of applicants at 13 cases. Median 3-year RFS was 14.5 months (95% CI 5.4-NA), median 3-year OS was not reached (95% CI 14.5-NA) and median3-year peritoneal RFS was 16.0 months (95% CI 5.4-NA). Median 3-year peritoneal RFS rate was 83% in CY0 and stamp+ cases (n=6), and 0% in CY1 and stamp+/- cases (n=7). (Log-rank p=0.015).

**Conclusion:**

Because of the slow accrual pace and early stop of the trial, we were not able to evaluate the prespecified endpoints thoroughly. However, EIPL might be effective to prevent perineal recurrence, especially in CY0 and stamp+ case.

## Introduction

Gastric cancer is the fifth most common malignant neoplasm and the third leading cause of cancer-related death in the world [1]. Peritoneal metastasis is one of the most frequent recurrence sites in patients who received gastrectomy for advanced gastric cancer, and this is associated with a quite poor survival outcome [2,3]. In gastric cancer, lavage cytology-positive (CY1) is related to peritoneal metastasis and the 5-year OS rate was reported to be only 2% in 1999 [4]. Due to development of chemotherapy, 5-year OS rate improved to 26% in a 2012 report [5]. Most recently, the 5-year OS rate for patients with CY1 or P1a was reported as 21.5–22.3%, which still remains unsatisfactory [6]. Peritoneal metastasis is developed by the implantation of peritoneal free cancer cells released from primary tumors [7,8]. Extensive intraoperative peritoneal lavage (EIPL) treatment has been attracting attention, because EIPL might be effective to prevent peritoneal metastasis by removing intraperitoneal free cancer cells before their fixation to peritoneum and progression.

A RCT conducted by Kuramoto revealed that the 5-year overall survival rate of patients receiving EIPL plus intraperitoneal cisplatin was much better than that of patients who received intraperitoneal cisplatin or no treatment after gastrectomy for CY1P0 gastric cancer [9]. EIPL can mechanically reduce the number of free cancer cells in the abdominal cavity through a limiting-dilution–like effect. While intraoperative or perioperative chemotherapy may exert a synergistic effect with EIPL by biologically eliminating cancer cells, EIPL alone might improve survival by removing intraperitoneal free cancer cells and preventing peritoneal metastasis. However, it remained unclear what kind of population is the best candidate of EIPL.

Our group revealed that the combination of intra-operative stamp cytology and peritoneal lavage cytology (CY) improved the identification of individuals with high risk of peritoneal metastasis [10]. The stamp cytology method can detect free cancer cells exposed on the serosal surface, which may lead to peritoneal metastasis in the near future. Accordingly, we considered that patients with CY1 and/or stamp+ could be the best candidate of EIPL, and started the current clinical trial in 2017.

On the other hand, in 2019, phase 3 RCT conducted by Misawa was reported, in which there is no statistically significant difference in DFS or OS between patients who had EIPL after gastrectomy for advanced gastric cancer of T3 status or above [11]. In addition, a clinical trial (JLSSG0901) revealed the non-inferiority of laparoscopic gastrectomy for advanced gastric cancer in 2023 [12]. This resulted in the significant decrease of open surgery cases. EIPL technically takes time in laparoscopic surgery. Due to the situations above, we decided to stop our trial, and analyze the already enrolled patients.

This study aimed to clarify the effectiveness of EIPL in patients with CY1 and/or stamp-positive findings. Although open surgery has become less common, making it premature to draw definitive conclusions from this study, it represents the first attempt to evaluate EIPL in this specific population. We believe that the data obtained will provide important insights for developing future treatment strategies for advanced gastric cancer.

## Materials and methods

### Study design

This study was a single-arm, multi-institutional, exploratory phase 2 trial conducted by Department of Gastroenterological Surgery, Osaka Metropolitan University Graduate School of Medicine. Between April 1, 2017, and March 31, 2021, patients enrolled from the following medical institutions: Department of Gastroenterological Surgery, Osaka Metropolitan University Graduate School of Medicine; Department of Gastroenterological Surgery, Osaka City General Hospital. Patients were planned to follow up until December 31, 2024.

In the stamp cytology technique, a glass slide was pressed against the serosal surface of the gastric tumor and subsequently stained using the Papanicolaou method. Cytological evaluation for the presence of cancer cells was performed by experienced cytopathologists. A “Stamp+” result was defined as the detection of cancer cells detected on the slides.

This study was performed according to the declaration of Helsinki and the Good Clinical Practice guidelines and was approved by the Medical Ethics Committee of Osaka Metropolitan University (approval no. 203664). Written informed consent was obtained from all participants before enrollment. The authors confirm that all ongoing and related trials for this intervention is registered. The registration number of UMIN-CTR is UMIN000059851.

Inclusion criteria before surgery were 1) histologically proven primary gastric adenocarcinoma (pap, tub1, tub2, por1, por2, sig, muc, special type) by upper gastrointestinal endoscopy, 2) possibility of achieving R0 or R1 resection (for CY1 and/ or stamp+) by gastrectomy with D2 lymphadenectomy, 3) esophageal invasion of 3 cm or less and no need for a thoracotomy, 4) age 20–80 years, 5) Eastern Cooperative Oncology Group (ECOG) performance status (PS) of 0 or 1, 6) no history of chemotherapy, endocrine therapy or radiotherapy, 7) ① 3,000/mm3 ≦ WBC ≦ 1,0000/mm3, ② Plt ≧ 100,000/mm3, ③ AST ≦ 100 IU/L, ALT ≦ 100 IU/L, ④ T-Bil ≦ 2.0 g/dl, ⑤ Cr ≦ 1.5 mg/dl, and 8) written informed consent from the patient.

### Peritoneal lavage cytology or stamp cytology

After laparotomy, the peritoneal lavage fluid was collected through conventional cytological methods with Papanicolaou staining as known as CY [13]. The serosal stamp sample from primary gastric tumor were collected during the operation as previously reported [10]. Sterilized slide glass was stamped to serosal wall of gastric cancer, and fixed with polyethylene glycol and ethanol (White fix; Yuai Kasei Co., Osaka, Japan). Fixed slide glasses were stained with Papanicolaou staining. Cytology assessment was performed by experienced cytopathologists without blinding.

### Extensive intraoperative peritoneal lavage treatment

After gastrectomy with D2 lymphadenectomy was performed, the peritoneal cavity was extensively shaken and washed, and then the fluid was completely aspirated. This procedure was repeated at least 12 times using 1 L of warm physiological saline. In total, 12 L or more of saline were to be used.

### Primary and secondary endpoints

The primary endpoint was relapse-free survival (RFS). Secondary endpoints were overall survival (OS), postoperative recurrence site and incidence of postoperative adverse events. RFS was defined as the interval between date of operation and recurrence of disease, including locoregional recurrence, distant metastasis or death from any cause. OS was defined as the interval between date of operation and death, irrespective of the cause. Postoperative recurrence site was diagnosed by CT scan. When there was suspicion of recurrence, additional imaging examination was performed to confirm the diagnosis as needed. Adverse events were defined as any complication classified by Clavien-Dindo classification. Adverse events were collected only until postoperative discharge, and their assessment was performed by the surgical team rather than by an independent adjudication committee. In addition, both EIPL-related and non-EIPL-related adverse events were included.

### Follow up

Adjuvant chemotherapy was performed based on the Japanese gastric cancer treatment guidelines at a physician’s discretion [14]. In principle, adjuvant therapy consisted of one year of S-1 monotherapy for patients with pStage II disease, whereas docetaxel plus S-1 (DS) therapy was administered to those with pStage III disease. During the 3 years after the operation, the survivors were assessed by physical examination and analysis of tumor markers every 3 months, and abdominal CT every 6 months.

### Patients recruitment

Sample size and patient recruitment based on our institutional experience, we estimated the median RFS for gastric cancer patients with CY1 and/or stamp to be 10.5 months (315 days). This value serves as the designated reference threshold for our study. We expected that the efficacy of the EIPL treatment would attain significance with an enhanced median RFS of approximately 13.9 months (418 days). With two-sided α at p < 0.05 and β at 0.20, the required sample size was 62, and assuming a possible drop-out, the target number of participants for enrollment was set at 65 patients.

The number of enrolled cases did not increase because of the results of other clinical trials mentioned in Introduction, and the principal investigator decided to stop the recruitment in December, 2022 at 13 cases enrollment after the discussion with research secretary and other researchers. In addition to the recruitment, the observation period is also shortened. As a result in the shortest case, the observation period was 29.8 months.

### Statistical analysis

The analysis was conducted on all eligible patients. Due to the limited sample size obtained, formal statistical analysis was not performed; instead, descriptive analyses were primarily employed. Perioperative electrolyte data are summarized as the mean (standard deviation), and changes from the preoperative score were assessed using paired t-tests. The length of Recurrence-Free Survival (RFS) and Overall Survival (OS) was described using Kaplan-Meier methods, and median values and percentages of relapse-free or survival patients were estimated. An exploratory subgroup analysis was performed by dividing the patients into two groups: CY0 and stamp+ cases, and CY1 and stamp + /- cases. Given the exploratory nature and limited sample size of this phase II study, inferential statistical tests were performed for descriptive purposes only, and p-values should be interpreted cautiously without formal hypothesis testing.

The analyses were conducted using JMP® Version 13 (SAS Institute Inc., Cary, NC).

## Results

### Patient characteristics

**Fig 1:**
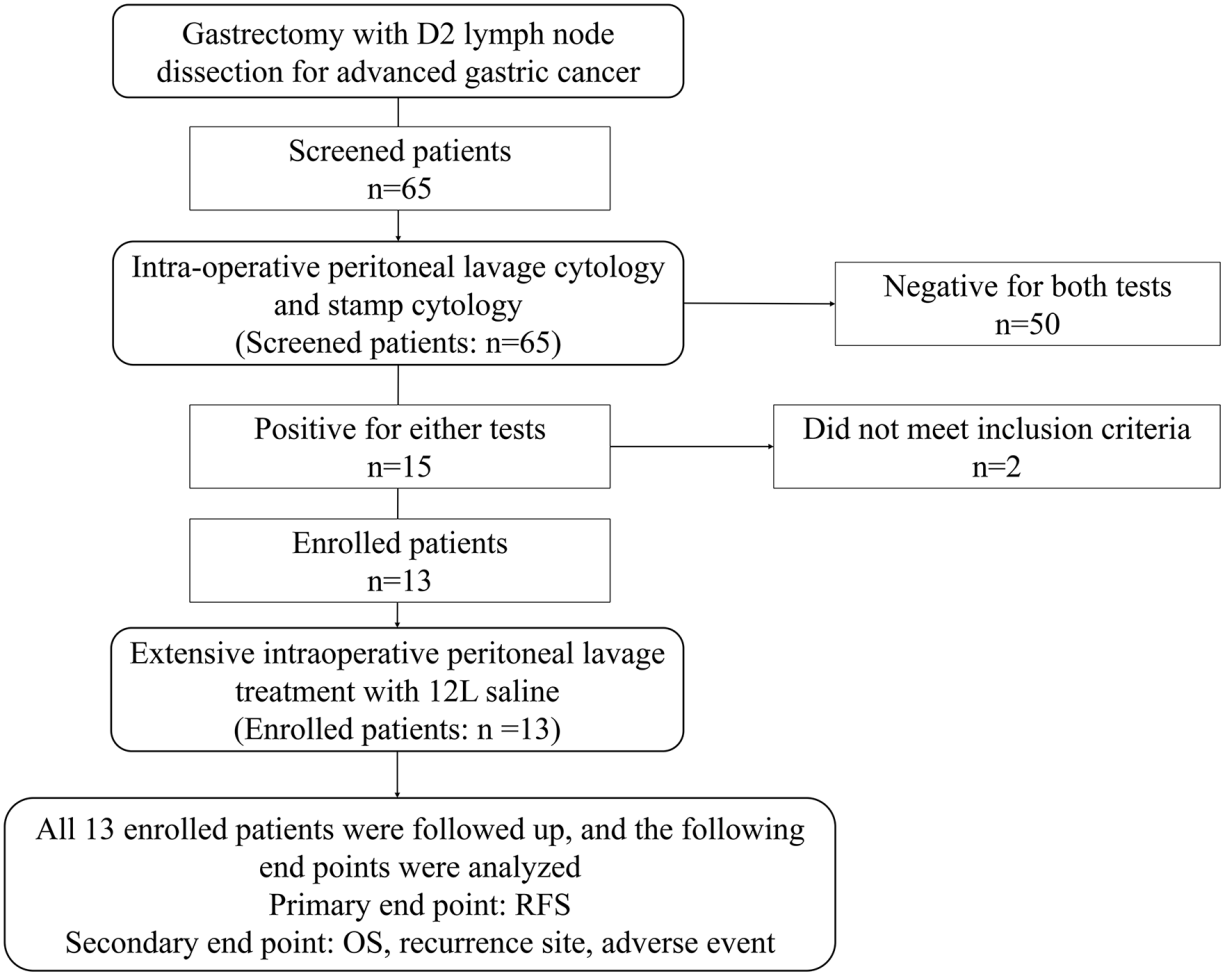
CONSORT diagram for the trial.

Between 2017 and 2021, We screened 65 patients who underwent curative gastrectomy with D2 lymphadenectomy. Among them, 13 patients from two institutions were enrolled in this study (Fig 1). The clinicopathologic characteristics and surgical outcomes were compared as shown in Table 1. Median follow-up for all patients was 29.8 months (Inter quartile range [IQR] 15.4–36). Distal gastrectomy was performed in 8 cases (61.5%), and total gastrectomy was performed in 5 cases (38.5%). D2 lymph node dissection was performed in all cases. CY1 was diagnosed in 7 cases (53.8%) and stamp + was diagnosed in 12 cases, which accounts for 92.3% in enrolled patients, and 17.6% in screened patients. CY1 and stamp + was diagnosed in six cases (46.2%), CY0 and stamp + was diagnosed in six cases (46.2%), and CY1 and stamp – was diagnosed in one case (7.7%).

**Table 1:** Patient clinicopathologic characteristics and surgical outcome.

		Total	
		n = 13	
Sex	female	5	38.5%
	male	8	61.5%
Average age (SD)		71.2(7.8)	
Average BMI		20.4(2.4)	kg/m^2^
CY/ Stump	CY1/ Stump+	6	46.2%
	CY1/ Stump-	1	7.7%
	CY0/ Stump+	6	46.2%
operative procedure	Distal gastrectomy	8	61.5%
	Total gastrectomy	5	38.5%
Lymph node dissection	D2	13	100.0%
Average surgical time (SD)		272.6(76.3)	min
Average blood loss (SD)		374(428.2)	ml
Radicality of resection	R0	6	46.2%
	R1	7	53.8%
	R2	0	0.0%
Histology	Differentiated	5	38.5%
	Undifferentiated	8	61.5%
pT	is, 1, 2	0	0.0%
	3	5	38.5%
	4a	8	61.5%
pN	0	0	0.0%
	1	4	30.8%
	2	2	15.4%
	3	7	53.8%
pStage	IIB	1	7.7%
	IIIA	3	23.1%
	IIIB	1	7.7%
	IIIC	1	7.7%
	IV	7	53.8%
Postoperative complication			
All complications	(+)	3	23.1%
	(-)	10	76.9%
Anastomotic leakage		1	7.7%
Abdominal abscess		1	7.7%
Pancreatic fistula		1	7.7%
hyponatremia		1	7.7%
Postoperative chemotherapy	(+)	11	84.6%
	(-)	2	15.4%

### Postoperative complication

Postoperative complications were confirmed in three cases; anastomotic leakage, abdominal abscess, pancreatic fistula and hyponatremia. And all cases were treated conservatively. Perioperative changes in average electrolyte levels are shown in Table 2. Serum sodium concentration (Na) on postoperative day one and three were significantly reduced from preoperative Na levels (p = 0.008, 0.007), and serum potassium concentration (K) on postoperative day one was significantly increased from preoperative K levels (p = 0.031). There was no significant change about serum cloride levels during the perioperative period.

**Table 2:** Perioperative changes in average electrolyte levels.

	Preoperative	POD1	p	POD3	p
Na (mEq/L)	140 (1.6)	135.2 (5.1)	0.008	136.5 (3.9)	0.007
K (mEq/L)	4.2 (0.6)	4.5 (0.5)	0.031	4.2 (0.4)	0.80
Cl (mEq/L)	104.6 (3.0)	103.6 (4.5)	0.49	103.9 (3.8)	0.57

### Survival

**Fig 2:**
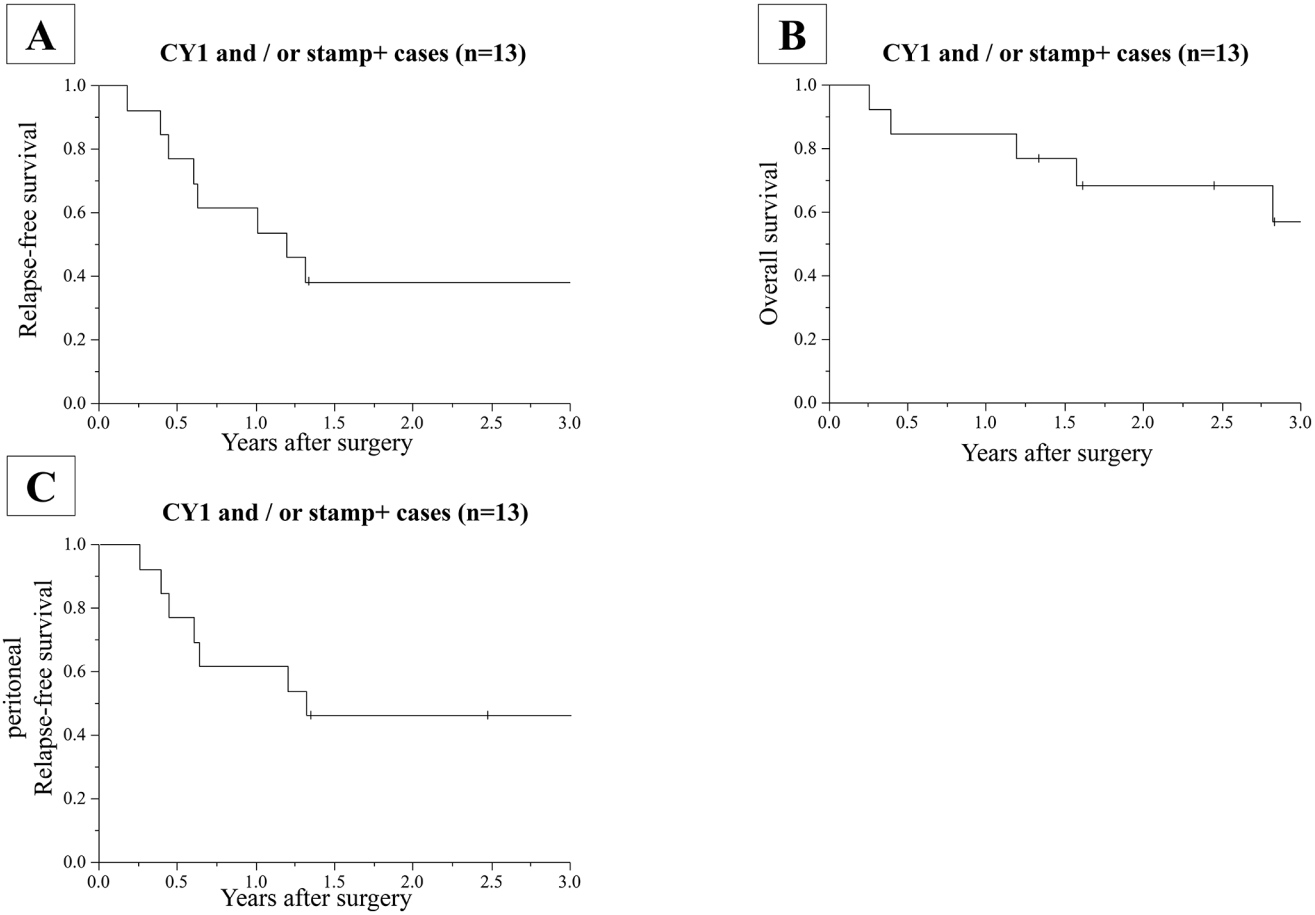
Kaplan–Meier analysis of Relapse-free survival, overall survival and peritoneal recurrence-free survival.

The 3-year RFS curve, OS curve and peritoneal RFS curve of the patients are shown in Fig 2. Median months to relapse was 14.5 months (95% CI 5.4-NA), and 3-year RFS rate was 38%. Median months to death was not estimated, and 3-year OS rate was 57% (Fig 2A, B).

The patterns of recurrence were shown in Table 3. Postoperative recurrence was confirmed in seven cases (53.8%). Peritoneal recurrence was confirmed in five cases (38.5%) and was the most common recurrent pattern. Four cases (30.8%) died from gastric cancer and one case died from another disease (COVID-19). Median months to peritoneal relapse was 16.0 months (95% CI 5.4-NA), and 3-year peritoneal RFS rate was 46% (Fig 2c).

**Table 3:** Site of first recurrence.

	n = 13	
Overall	7	53.8%
Peritoneum	5	38.5%
Lymph node	2	15.4%
Lung	1	7.7%

### Subgroup analysis

We separately analyzed the following two groups: CY0 and stamp+ cases (n = 6), and CY1 and stamp + /- cases (n = 7).

**Fig 3:**
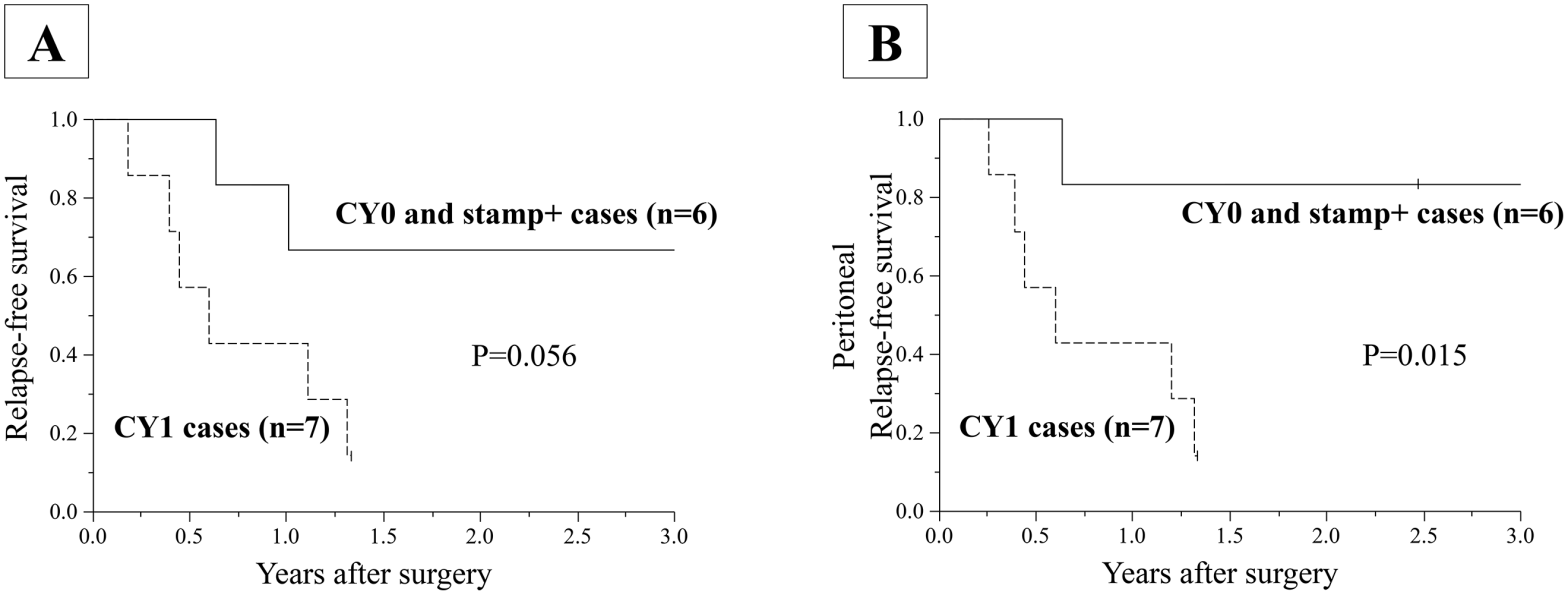
Kaplan–Meier analysis of Relapse-free survival and peritoneal recurrence-free survival for the stratified analyses.

3-year RFS rate was 67% in CY0 and stamp+ cases (n = 6), and 0% in CY1 and stamp + /- cases (n = 7). There was a borderline significant difference between two groups in 3-year RFS (p = 0.056, log-rank test) (Fig 3A). 3-year peritoneal RFS rate was 83% in CY0 and stamp+ cases (n = 6), and 0% in CY1 and stamp + /- cases (n = 7). As for the 3-year peritoneal PFS, CY0 and stamp+ cases showed significantly better survival outcome than CY1 and stamp + /- cases (p = 0.015, log-rank test) (Fig 3B).

## Discussion

Because of the recent decline in the number of open abdominal surgery, we need to stop the trial at 13 cases enrollment. As shown in Fig 2A, B, Median RFS was 14.5 months (3-year RFS was 38%) and median OS was NR months (3-year OS was 57%), these results were relatively good compared with our prespecified endpoints, although we were not able to analyze the endpoints appropriately due to the lack of required cases. This suggests that EIPL might be effective to prevent peritoneal recurrence in selected cases.

Although the subgroup analysis suggested that EIPL may preferentially reduce peritoneal recurrence in biologically selected patients—particularly those with CY0 and stamp-positive disease—these findings are exploratory in nature. Because the primary subgroup result for peritoneal RFS was derived from a very small sample, the survival estimates are inherently unstable, increasing the vulnerability of the study to type I error. Therefore, these observations should be interpreted with caution, and validation in adequately powered, controlled trials will be essential to determine whether EIPL truly confers a peritoneal recurrence–reducing benefit in these specific patient populations.

Recently, impact of EIPL for prevention of peritoneal dissemination from gastric cancer has been examined. Misawa and colleagues evaluated the effect of EIPL for gastric cancer with cT3-4 [11], and they concluded that EIPL did not improve survival for this population. However, this result may be because the most patients in their study had negative findings on CY. Furthermore, although cT3-4 status in pre-operative diagnosis was inclusion criteria in their study, 18.0% of cases were diagnoses as pT1-2 postoperatively. The selection of peritoneal metastasis high risk patients should be critical to evaluate the effect of EIPL, but as mentioned above, only with preoperative imaging, accurate tumor depth diagnosis is difficult. On the other hand, our group reported that intra-operative stamp cytology can identify high risk of peritoneal metastasis [10]. In this study, some of pT3 cases were diagnosed as stamp + . We previously reported that the distance from the tumor invasion front to the serosa (DIFS) is useful for predicting peritoneal metastasis [15]. A positive stamp cytology result theoretically indicates a shorter DIFS. Therefore, we consider stamp cytology to be a clinically useful technique for identifying patients with a small DIFS (or those who are essentially T4a), who are at particularly high risk for peritoneal metastasis.

In this research, in order to recruit patients at high risk of peritoneal dissemination, we determined to include cases who are diagnosed as CY1 and/ or stamp + . Indeed, postoperative recurrence with perineal metastasis was observed in five cases (38.5%) and it was the most common recurrence site in this study. Depth of invasion were deeper than T3 in all cases. Accordingly, inclusion criteria we set seems to be appropriate in terms of the risk of peritoneal metastasis. In the subgroup analysis, the 3-year RFS of CY0 and stamp+ cases were 66.7%. Although the analyzed number of patients was small, this data is similar to the 3-year RFS rate of 67.7% of stage III gastric cancer in JACCRO GC-07 trial with the S-1 plus docetaxel (DS) group [16]. Considering that only half of stamp+ cases underwent DS treatment as adjuvant, EIPL might improve survival outcome of patients who did not undergo DS treatment. However, we cannot definitely conclude the effect of EIPL, since we did not compare the results between EIPL and non-EIPL group. In addition, DS therapy was not balanced between groups and no adjustment or stratification was performed; therefore, it may have acted as a confounding factor. As a result, our speculation that EIPL might have compensated for the limited use of DS therapy should be interpreted with caution. On the other hand, what we can conclude is that CY0, Stamp+ cases showed significantly better peritoneal recurrence free survival than CY1 cases. This data implies that the impact of stamp+ should be different from CY1, and rather similar to pT4a. This is quite important finding, and stamp cytology could be useful in future clinical study, when researchers would like to select only pT4a cases during operation.

Intraperitoneal chemotherapy (IPC) may be alternative option for decreasing peritoneal recurrence for high risk patients, although the evidence about IPC has not been definite. Kuramoto et al. demonstrated that IPC combined with EIPL was superior to surgery plus EIPL or surgery alone in patients with positive lavage cytology. The Phoenix GC trial indicated that IPC was promising for patients with Stage IV, although its superiority was not statistically proven. According to these data, IPC could lead to possible benefit of IPC as biological irrigation, instead of EIPL. In addition, it remains debatable whether normal saline is the best solution for EIPL. Akasaka et al reported that lactate Ringer’s solution is the better solution for reducing the risk of peritoneal metastasis. This issue should be investigated in future [17].

Complications related to EIPL was confirmed in one case; hyponatremia. After the treatment only with replenishment of electrolytes, hyponatremia was improved promptly. The other complications related to EIPL didn’t develop. Although perioperative electrolyte changes were statistically significant, the actual amount of change is not so much. In addition, it is suggested that secretion of ADH due to surgical stress may cause hyponatremia [18]. EIPL was performed without serious complications; however, the single-arm design and absence of a control group limit our ability to conclude its safety or attribute survival outcomes to EIPL.

There are several limitations to this study. First, the number of enrolled patients was too small. The extremely small sample size resulted in highly unstable survival estimates, which undermines the reliability of the findings. This limitation should be taken into account when interpreting the results. We considered several reasons for this slow enrollment. As described above, the number of laparoscopic surgeries increased during the study period. Although EIPL can be performed during laparoscopic surgery, stamp examination is technically difficult in the laparoscopic setting. Considering the potential benefits of stamp cytology, it may be necessary to develop new devices, such as small but durable slide glasses that can be introduced into the abdominal cavity through laparoscopic ports. Second, the number of patients with gastric cancer itself has been decreasing, largely due to the eradication of *Helicobacter pylori*. Finally, the COVID-19 pandemic also contributed to the reduced number of surgical procedures. In addition, although this was a prospectively designed study, some post-hoc survival analyses were performed, and the statistical power to detect differences between groups was clearly insufficient. Furthermore, because the study was terminated pragmatically rather than according to predefined stopping rules or an interim analysis plan, some degree of bias may have been introduced. This limitation should be taken into account when interpreting the results.

Although definite conclusion could not be drawn due to the lack of the required number of cases, we were able to perform EIPL safely, and EIPL might be effective for selective patients. Stamp cytology should be effective to diagnose real T4a cases intraoperatively, and useful technique for selecting T4a cases in future clinical trials.

## Supporting information

S1 File Minimal data set. (XLSX)

S2 File Study protocol (English). (DOCX)

S3 File Study protocol (Japanese). (DOCX)

S4 File TREND checklist. (DOCX)
