# Supplementary material for: Extensive intraoperative peritoneal lavage (EIPL) for gastric cancer with positive peritoneal lavage and/or stamp cytology: An exploratory phase II study
Source: PLoS One. 2026 Apr 17;21(4):e0347742. doi: 10.1371/journal.pone.0347742 (PMC13089869; doi:10.1371/journal.pone.0347742)
Supplement: S2 File — (DOCX) [file pone.0347742.s002.docx]

**A phase II study to evaluate the intraoperative peritoneal lavage treatment for patients who are diagnosed as positive by peritoneal lavage cytology or stamp cytology**

**Research Protocol**

**Principal Investigator**

**Masaichi Ohira, M.D., Ph.D.**
Department of Surgical Oncology,
Graduate School of Medicine, Osaka City University
1-4-3 Asahi-machi, Abeno-ku, Osaka 545-8585, Japan
Tel: +81-6-6645-3838 Fax: +81-6-6646-6450
E-mail: masaichi@med.osaka-cu.ac.jp

**Administrative Office**

**Yuichiro Miki, M.D., Ph.D.**
Department of Surgical Oncology / Molecular Oncology and Therapeutics,
Graduate School of Medicine, Osaka City University
1-4-3 Asahi-machi, Abeno-ku, Osaka 545-8585, Japan
Tel: +81-6-6645-3838 Fax: +81-6-6646-6450
E-mail: y_miki@med.osaka-cu.ac.jp

**Masakazu Yashiro, M.D., Ph.D.**Department of Surgical Oncology / Molecular Oncology and Therapeutics,
Graduate School of Medicine, Osaka City University
1-4-3 Asahi-machi, Abeno-ku, Osaka 545-8585, Japan
Tel: +81-6-6645-3838 Fax: +81-6-6646-6450
E-mail: m9312510@med.osaka-cu.ac.jp

**First Edition:** December 27, 2016
**Approved by:** Institutional Review Board,
Graduate School of Medicine, Osaka City University
**Date of Approval:** December 28, 2016
**Approval Number:** 203664

# **Overview**

## **Schema**

Patients scheduled to undergo gastrectomy with D2 lymph node dissection for advanced gastric cancer will be enrolled.

・Age between 20 and 80 years / ・Performance status (PS) of 0 or 1

Written informed consent will be obtained preoperatively

Patient Registration

Patients with negative intraoperative peritoneal cytology will be excluded.

During surgery, peritoneal lavage cytology and

stamp cytology will be performed.

Patients with a positive result in any of the intraoperative cytology tests will be eligible for enrollment.

During surgery, before abdominal closure,

the peritoneal cavity will be lavaged with 12 liters of normal saline.

Follow-up period: 3 years postoperatively

<<B群

手術中、閉腹前に2Lの生理食塩水で腹腔内洗浄

## **Background**

- Peritoneal recurrence is common even in patients with **gastric cancer who have undergone curative resection**.
- **Peritoneal lavage cytology** has traditionally been used to predict peritoneal recurrence; however, its **low sensitivity** remains a concern. Previous studies at our institution have shown that **adding imprint cytology** can improve the sensitivity of detection.
- Typically, the peritoneal cavity is lavaged with approximately **2 liters of normal saline** after gastrectomy. Some reports suggest that **extensive intraoperative peritoneal lavage with normal saline** in patients with positive peritoneal cytology may improve survival outcomes.
- However, to date, **no prospective studies** have investigated the impact of extensive intraoperative peritoneal lavage on **recurrence-free survival** in patients with positive peritoneal or imprint cytology.

## **Objectives**

　This non-randomized Phase II study aims to evaluate the efficacy of extensive intraoperative peritoneal lavage on recurrence-free survival in patients scheduled to undergo gastrectomy with D2 lymph node dissection who have positive intraoperative peritoneal cytology or positive gastric wall imprint cytology.

In addition, data regarding safety and feasibility will be collected as secondary endpoints.

Primary endpoint: Recurrence-free survival

Secondary endpoints: ① Overall survival, ② Sites of postoperative recurrence, ③ Incidence of postoperative adverse events

## **Patients**

1. Histologically confirmed gastric cancer from the primary gastric lesion by endoscopic biopsy, including one of the following types: papillary (pap), well-differentiated tubular (tub1), moderately differentiated tubular (tub2), poorly differentiated (por1, por2), signet-ring cell (sig), mucinous (muc), or other special types.
2. Positive intraoperative peritoneal cytology or gastric wall imprint cytology, with no evidence of distant metastasis. Patients must be candidates for R0/1 gastrectomy with D2 lymph node dissection (total or distal gastrectomy, including laparoscopic procedures). General anesthesia with or without epidural anesthesia will be used.
3. Esophageal invasion of 3 cm or less, with no thoracotomy required.
4. Age between 20 and 80 years.
5. ECOG performance status of 0 or 1.
6. No prior chemotherapy (including endocrine therapy) or radiotherapy for other malignancies.
7. Adequate organ function, confirmed within 56 days prior to enrollment (tests performed on the same day of the week up to 8 weeks before enrollment are acceptable):

- White blood cell count: 3,000–10,000 /mm³
- Platelet count ≥ 100,000 /mm³
- AST ≤ 100 IU/L, ALT ≤ 100 IU/L
- Total bilirubin ≤ 2.0 mg/dL
- Serum creatinine ≤ 1.5 mg/dL

1. Written informed consent obtained from the patient.

## **Treatment**

Surgery will be performed according to the Japanese Gastric Cancer Treatment Guidelines (3rd edition, for physicians), including D2 or more extensive lymph node dissection.

During the operation, the peritoneal cavity will be lavaged with 12 liters of normal saline prior to abdominal closure.

## **Planned Sample Size and Study Duration**

- Planned number of eligible patients: 65
- Enrollment period: 2 years
- Follow-up period: 3 years after completion of enrollment
- Total study duration: 5 years

## **Contact Information**

**Administrative Office**

**Yuichiro Miki, M.D., Ph.D.**
Department of Surgical Oncology / Molecular Oncology and Therapeutics,
Graduate School of Medicine, Osaka City University
1-4-3 Asahi-machi, Abeno-ku, Osaka 545-8585, Japan
Tel: +81-6-6645-3838 Fax: +81-6-6646-6450
E-mail: y_miki@med.osaka-cu.ac.jp

**Masakazu Yashiro, M.D., Ph.D.**
Department of Surgical Oncology / Molecular Oncology and Therapeutics,
Graduate School of Medicine, Osaka City University
1-4-3 Asahi-machi, Abeno-ku, Osaka 545-8585, Japan
Tel: +81-6-6645-3838 Fax: +81-6-6646-6450
E-mail: m9312510@med.osaka-cu.ac.jp

**Table of Contents**

[**0** **Overview** i](#_Toc212196656)

[**Schema** i](#_Toc212196657)

[**0.1** **Background** i](#_Toc212196658)

[**0.2** **Objectives** i](#_Toc212196659)

[**0.3** **Patients** ii](#_Toc212196660)

[**0.4** **Treatment** ii](#_Toc212196661)

[**0.5** **Planned Sample Size and Study Duration** iii](#_Toc212196662)

[**0.6** **Contact Information** iii](#_Toc212196663)

[**1** **Background** 1](#_Toc212196664)

[**1.1** **Significance of This Study** 1](#_Toc212196665)

[**1.2** **Ancillary Studies** 1](#_Toc212196666)

[**2** **Overview of Investigational Product** 2](#_Toc212196667)

[**2.1** **Investigational Product** 2](#_Toc212196668)

[**2.2** **Expected Adverse Reactions and Device Malfunctions** 2](#_Toc212196669)

[**3** **Diagnostic Criteria, Staging, and Classification of Target Disease** 2](#_Toc212196670)

[**3.1** **Gastric Cancer Guidelines** 2](#_Toc212196671)

[**3.2** **Performance Status (PS) Assessment** 2](#_Toc212196672)

[**4** **Eligible Patients** 2](#_Toc212196673)

[**4.1** **Inclusion Criteria** 2](#_Toc212196674)

[**4.2** **Exclusion Criteria^9^** 3](#_Toc212196675)

[**5** **Patient Registration** 4](#_Toc212196676)

[**5.1** **Registration Procedure** 4](#_Toc212196677)

[**6** **Study Design** 4](#_Toc212196678)

[**6.1** **Type and Design** 4](#_Toc212196679)

[**6.2** **Clinical Hypothesis and Sample Size Determination** 4](#_Toc212196680)

[**6.3** **Accrual Plan** 5](#_Toc212196681)

[**6.4** **Outline of the Study** 6](#_Toc212196682)

[**6.5** **Study Participation Period for Subjects** 6](#_Toc212196683)

[All subjects will be followed up for 36 months postoperatively. Follow-up will be conducted at outpatient visits every 3 months (maximum 12 visits within 36 months). 6](#_Toc212196684)

[**6.6** **Dosage, Administration, and Duration of Study Drug** 6](#_Toc212196685)

[**6.7** **Regulations on Concomitant Medications (Therapies)** 6](#_Toc212196686)

[**6.8** **Handling and Dispensing of Study Drug** 6](#_Toc212196687)

[**6.9** **Post-Trial Care** 6](#_Toc212196688)

[After the study, efforts will be made to ensure that subjects receive the best possible preventive, diagnostic, and therapeutic care based on the results of the study. 6](#_Toc212196689)

[**6.10** **Storage and Use of Samples for Other Institutions** 6](#_Toc212196690)

[**7** **Observations and Examinations, Schedule** 7](#_Toc212196691)

[**7.1** **8-1　Pre-Registration Assessment Items** 7](#_Toc212196692)

[**7.2** **Intraoperative and Postoperative Assessment Items** 7](#_Toc212196693)

[**8** **Criteria for Discontinuation of Individual Subjects** 9](#_Toc212196694)

[**9** **Handling of Adverse Events** 9](#_Toc212196695)

[**9.1** **Response to Subjects for Adverse Events** 9](#_Toc212196696)

[**9.2** **Reporting of Serious Adverse Events** 10](#_Toc212196697)

[**10** **Study Completion, Discontinuation, or Interruption** 10](#_Toc212196698)

[**10.1** **Study Completion** 10](#_Toc212196699)

[**10.2** **Discontinuation or Interruption of the Study** 10](#_Toc212196700)

[**11** **Endpoints** 11](#_Toc212196701)

[**11.1** **Primary Endpoint** 11](#_Toc212196702)

[**11.2** **Secondary Endpoints** 11](#_Toc212196703)

[**12** **Data Collection** 11](#_Toc212196704)

[**12.1** **Case Report Forms (CRF)** 11](#_Toc212196705)

[**12.2** **Document Storage** 12](#_Toc212196706)

[**13** **Statistical Analysis** 12](#_Toc212196707)

[**13.1** **Data Handling** 12](#_Toc212196708)

[**13.2** **Efficacy Analysis** 12](#_Toc212196709)

[**13.3** **Safety Analysis** 12](#_Toc212196710)

[**14** **Trial Period** 13](#_Toc212196711)

[**15** **Ethical Considerations** 13](#_Toc212196712)

[**15.1** **Compliance with Guidelines and Declaration of Helsinki** 13](#_Toc212196713)

[**15.2** **Ethics Committee Approval** 13](#_Toc212196714)

[**15.3** **Provision of Explanation Documents and Information to Subjects** 13](#_Toc212196715)

[**15.4** **Consideration for Human Rights (Protection of Personal Information)** 14](#_Toc212196716)

[**15.5** **Consideration for Safety and Disadvantages** 14](#_Toc212196717)

[**15.6** **Handling of Inquiries** 14](#_Toc212196718)

[**16** **Study Costs** 14](#_Toc212196719)

[**16.1** **Study Funding and Conflicts of Interest** 14](#_Toc212196720)

[**16.2** **Patient Cost Burden** 14](#_Toc212196721)

[**16.3** **Compensation and Insurance for Health Damage** 15](#_Toc212196722)

[**17** **Record Retention** 15](#_Toc212196723)

[**18** **Trial Registration and Publication of Results** 15](#_Toc212196724)

[**19** **Study Organization** 15](#_Toc212196725)

[**Principal Investigator** 15](#_Toc212196726)

[**19.1** **Participating Facilities & Principal Investigators** 15](#_Toc212196727)

[**19.2** **Administrative Office** 16](#_Toc212196728)

[**19.3** **Safety and Efficacy Evaluation Committee** 16](#_Toc212196729)

[**19.4** **Monitoring Officer** 16](#_Toc212196730)

[**19.5** **Biostatistician** 16](#_Toc212196731)

[**20** **Protocol Deviations or Amendments** 17](#_Toc212196732)

[**21** **Monitoring / Data Quality Management** 17](#_Toc212196733)

[**22** **Audits** 17](#_Toc212196734)

[**23** **References** 18](#_Toc212196735)

# **Background**

The mortality rate of gastric cancer in Japan has continued to decline in both men and women since the 1970s, and in men, lung cancer surpassed gastric cancer as the leading cause of cancer-related death after 1993; however, gastric cancer remains the second leading cause of death from malignant neoplasms in Japan¹. According to the Japanese Gastric Cancer Treatment Guidelines (3rd edition), standard surgery for tumors with N(+) involvement or T2 or deeper is either distal gastrectomy or total gastrectomy. Total gastrectomy is indicated when an adequate resection margin cannot be secured by distal gastrectomy. Regarding lymph node dissection for advanced gastric cancer, based on the results of the JCOG9501 trial conducted in Japan, D2 lymphadenectomy is considered the standard treatment. Concerning adjuvant therapy after curative resection, based on the results of the ACTS-GC trial (Adjuvant Chemotherapy Trial of S-1 for Gastric Cancer), postoperative administration of S-1 for one year has become the standard treatment in Japan for patients with stage II–IIIB gastric cancer (excluding pT1)². Furthermore, following the results of the randomized phase III trial (CLASSIC trial) comparing surgery alone with adjuvant chemotherapy using capecitabine plus oxaliplatin (CapeOX), CapeOX therapy is also recommended as a postoperative adjuvant chemotherapy option³.
 These treatment developments have improved the survival outcomes of patients undergoing gastric cancer surgery. However, even after curative resection, peritoneal recurrence has been reported in 37–39.4% of cases (JCOG9206-1, JCOG9206-2, JCOG9501 trials). In all these trials, the most common site of recurrence after curative resection was the peritoneum. In the ACTS-GC trial, peritoneal recurrence occurred in 14.6% (77/549) of patients even with adjuvant chemotherapy⁴. These findings indicate that even among cases currently judged as curatively resected by staging, there are high-risk patients for peritoneal dissemination recurrence.
 At present, peritoneal cytology (CY) is the most widely used method to predict peritoneal dissemination recurrence. The 5-year overall survival rate for CY-positive cases has been reported to be approximately 0–2%, indicating an extremely poor prognosis. This highlights the need for novel treatment development to improve outcomes in this patient population^⁵,⁶^.
However, the current problem with CY is its low sensitivity in predicting recurrence. In a previous study conducted at our institution, the sensitivity increased from 25.0% to 47.1% when imprint cytology was used in combination with standard CY. Considering the importance of including as many high-risk patients as possible in novel treatment development, it is necessary to advance new therapies even for patients who are positive on imprint cytology.
 In the past, small-scale randomized controlled trials have been conducted targeting CY-positive cases to examine the efficacy of gastrectomy combined with extensive intraoperative peritoneal lavage (EIPL) and intraperitoneal chemotherapy⁷. Among these, the combination of EIPL and intraperitoneal chemotherapy showed the best outcomes, but even EIPL alone was more effective than surgery alone. Furthermore, in single-arm studies, EIPL has been reported to achieve a 5-year survival rate of 46.5% in CY1P0 cases, suggesting that EIPL alone may contribute to recurrence prevention⁸. However, no prospective trial has examined the effect of EIPL on survival in CY-positive or imprint cytology-positive cases.
 In previous reports, 10 liters of peritoneal lavage were performed. At our institution, 2 liters are typically used at the end of surgery. Therefore, in this trial, we set the intervention to evaluate the effect of an additional 10 liters of lavage (total 12 liters) compared with the standard method. Performing extensive intraoperative lavage immediately before the end of surgery is unlikely to cause severe adverse events, and this intervention can be easily implemented regardless of the institution. Based on these considerations, we designed a non-randomized phase II trial to evaluate the superiority of 12-liter peritoneal lavage in terms of recurrence-free survival in patients who are CY-positive or imprint cytology-positive.

## **Significance of This Study**

The standard treatment for advanced gastric cancer is gastrectomy with D2 lymphadenectomy followed by adjuvant chemotherapy; however, the frequency of postoperative recurrence remains substantial, highlighting the need for further therapeutic development. In particular, overcoming the high incidence of peritoneal recurrence is a critical challenge, and evaluating the efficacy of extensive intraoperative peritoneal lavage in this study is therefore considered meaningful.

## **Ancillary Studies**

In this study, subgroup analyses are planned for patients with positive peritoneal cytology and positive imprint cytology. Additionally, since intraoperative blood loss and transfusion volume have been reported to be associated with recurrence, these data will also be examined. Samples obtained by scraping the gastric wall during surgery will be used to investigate the relationship between gene expression in the scraped cells, clinicopathological factors, and prognosis.
 For any future ancillary studies using data from this trial, the proposed study plan must be documented and submitted to the principal investigator. The principal investigator will review the proposed study content and methodology together with the proposer.

# **Overview of Investigational Product**

## **Investigational Product**

Normal saline (sodium 154 mEq/L, chloride 154 mEq/L) will be used.

## **Expected Adverse Reactions and Device Malfunctions**

No adverse events have been reported with intraperitoneal use of the above solution. However, large intravenous doses may cause electrolyte disturbances, congestive heart failure, edema, or acidosis.

# **Diagnostic Criteria, Staging, and Classification of Target Disease**

## **Gastric Cancer Guidelines**

Terminology and classification in this protocol follow the 14th edition of the Japanese Gastric Cancer Classification and the 3rd edition of the Japanese Gastric Cancer Treatment Guidelines.

## **Performance Status (PS) Assessment**

The Japanese translation of the ECOG scale will be used:

| Grade | | Performance Status |
| --- | --- | --- |
| 0 | Asymptomatic, fully active, able to carry out all pre-disease activities without restriction. | |
| 1 | Mild symptoms, restricted in physically strenuous activity but ambulatory and able to do light work (e.g., light housework, office work). | |
| 2 | Ambulatory and capable of self-care but unable to perform work activities; up >50% of waking hours. | |
| 3 | Limited self-care, often requires assistance; confined to bed/chair >50% of waking hours. | |
| 4 | Completely disabled, requires constant care, confined to bed. | |

# **Eligible Patients**

Patients who meet all inclusion criteria and none of the exclusion criteria will be considered eligible. Staging and histological classification follow the 14th edition of the Japanese Gastric Cancer Classification.

## **Inclusion Criteria**

1) Histologically confirmed gastric cancer by endoscopic biopsy (pap, tub1, tub2, por1, por2, sig, muc, or special types).

2) Positive peritoneal cytology or imprint cytology, no other distant metastasis, and R0/1 resection feasible via D2 gastrectomy (total or distal, including laparoscopic), under general ± epidural anesthesia.

3) Esophageal invasion ≤3 cm; no thoracotomy required.

4) Age 20–80 years.

5) ECOG PS 0–1.

6) No prior chemotherapy (including endocrine therapy) or radiotherapy for other cancers.

7) All of the following criteria must be met. (The latest preoperative laboratory values within 56 days before enrollment will be used. Tests performed on the same day of the week 8 weeks prior to enrollment are acceptable.)

① White blood cell count: 3,000–10,000/mm³

② Platelet count ≥100,000/mm³

③ AST ≤100 IU/L, ALT ≤100 IU/L

④ Total bilirubin ≤2.0 mg/dL

⑤ Serum creatinine ≤1.5 mg/dL

8) Written informed consent for study participation has been obtained from the patient.

［Rationale］

1) To ensure that the case is histologically confirmed as gastric cancer.

2) Patients with positive peritoneal cytology or imprint cytology are considered high-risk for peritoneal recurrence and are therefore eligible. However, patients with other distant metastases are often unable to achieve R0/1 resection and are excluded from this study.

3) Patients with esophageal invasion ≥3 cm may require thoracotomy and are excluded.

4 -7) Special cases or patients with poor preoperative general condition are excluded.

8) Patient consent is mandatory.

## **Exclusion Criteria**[**^9^**](#_ENREF_9)

1) Pregnant or breastfeeding women.

2) Psychiatric disorders interfering with participation.

3) Continuous systemic steroid use.

4) Myocardial infarction within 6 months or unstable angina.

5) Uncontrolled hypertension.

6) Insulin-treated or uncontrolled diabetes.

7) Respiratory disease requiring continuous oxygen.

［Rationale］

1)〜7) To ensure the validity of efficacy evaluation and to consider patient safety. These criteria were set in accordance with previous clinical trials in gastric cancer cases.[^9^](#_ENREF_9)

# **Patient Registration**

## **Registration Procedure**

**Eligible Patients:** Prior to surgery, the principal investigator or sub-investigator will obtain written informed consent. If the patient meets all inclusion criteria (except criterion 2) and none of the exclusion criteria, the case registration form will be completed and the patient will be enrolled in the study.

**Analysis Population:** If intraoperative findings confirm that the patient meets the eligibility criteria, the eligibility confirmation form will be completed, and the protocol treatment will be administered. The case registration form and eligibility confirmation form will be stored at the study office. Any withdrawal of consent, discontinuation, or dropout must be reported promptly.

**Administrative Office**

**Yuichiro Miki, M.D., Ph.D.**
Department of Surgical Oncology / Molecular Oncology and Therapeutics,
Graduate School of Medicine, Osaka City University
1-4-3 Asahi-machi, Abeno-ku, Osaka 545-8585, Japan
Tel: +81-6-6645-3838 Fax: +81-6-6646-6450

**Masakazu Yashiro, M.D., Ph.D.**Department of Surgical Oncology / Molecular Oncology and Therapeutics,
Graduate School of Medicine, Osaka City University
1-4-3 Asahi-machi, Abeno-ku, Osaka 545-8585, Japan
Tel: +81-6-6645-3838 Fax: +81-6-6646-6450

# **Study Design**

## **Type and Design**

This is a single-arm, non-randomized phase II study to evaluate the superiority of extensive intraoperative peritoneal lavage for recurrence-free survival in patients undergoing D2 gastrectomy with positive intraoperative peritoneal or imprint cytology. For setting the threshold, survival outcomes of 138 eligible patients operated at our department from 1997 to 2012 were used.

**Rationale:** The target population is rare, and patient accrual may require considerable time. Therefore, a randomized controlled trial is considered difficult, and this single-arm phase II study uses historical outcomes to set thresholds.

## **Clinical Hypothesis and Sample Size Determination**

1. **Clinical Hypothesis:**

Extensive intraoperative peritoneal lavage may prolong recurrence-free survival in patients with positive intraoperative peritoneal or imprint cytology after D2 gastrectomy.

1. **Sample Size Determination:**

The median recurrence-free survival of CY- or imprint-positive patients operated at our hospital from 1997 to 2012 was 315 days (0.86 years). This value was set as the threshold, and the expected median RFS with the study intervention is 438 days (1.2 years). Assuming α=0.05 and β=0.20, 62 patients are required. The planned enrollment is 65 patients.

## **Accrual Plan**

In 2011, 20 patients met eligibility criteria at Osaka City University Hospital. Assuming a 75% consent rate, 15 patients per year are expected. Single-center accrual is insufficient for timely completion; therefore, this study will be conducted as a multicenter trial. With participation from institutions expected to enroll 5–15 patients per year, total annual accrual is estimated at approximately 35 patients. The enrollment period will be 2 years. A 3-year follow-up period after enrollment completion is required, making the total study duration 5 years.

## **Outline of the Study**

| Consent acquisition | ⇨ | Registration | ⇨ | Preoperative examination at the start of surgery | ⇨ | Eligibility confirmation |  |  |  | Postoperative follow-up for 3 years Blood tests (every 3 months) CT (every 6 months) Upper gastrointestinal endoscopy (once a year) |
| --- | --- | --- | --- | --- | --- | --- | --- | --- | --- | --- |
|  |  |  |  |  |  |  | ⇨ |  |  |  |
|  |  |  |  |  |  |  |  | Intraoperative extensive peritoneal lavage at the end of surgery |  |  |
|  |  |  |  |  |  |  | ⇨ |  |  |  |
|  |  |  |  |  |  |  |  |  |  |  |

## **Study Participation Period for Subjects**

## All subjects will be followed up for 36 months postoperatively. Follow-up will be conducted at outpatient visits every 3 months (maximum 12 visits within 36 months).

## **Dosage, Administration, and Duration of Study Drug**

Intraoperative extensive peritoneal lavage will be performed using 12 L of physiological saline at the end of surgery.

## **Regulations on Concomitant Medications (Therapies)**

1) Concomitant medications (therapies): None

2) Prohibited concomitant medications (therapies): None

## **Handling and Dispensing of Study Drug**

The drugs used in this study are those routinely stocked in the surgical department.

## **Post-Trial Care**

## After the study, efforts will be made to ensure that subjects receive the best possible preventive, diagnostic, and therapeutic care based on the results of the study.

## **Storage and Use of Samples for Other Institutions**

Samples obtained for imprint cytology in relation to this study will be stored in the Department of Molecular Oncology for 5 years after the study report is completed. The study office is responsible for management and will report the management status to the hospital director. Disposal of samples will be conducted in an anonymized state.
 Researchers will be instructed and supervised to ensure the accuracy of information and samples obtained from humans, which will also be stored for 5 years after study completion under secure management to prevent leakage, mix-up, theft, or loss.

# **Observations and Examinations, Schedule**

## **8-1　Pre-Registration Assessment Items**

**To be performed within 56 days prior to registration:**

1) General condition: PS (ECOG), height, weight

2) Peripheral blood count: WBC, hemoglobin, platelet count

3) Blood biochemistry: AST, ALT, total bilirubin, creatinine, CRP

4) Tumor markers: CEA, CA19-9

5) Contrast-enhanced CT of upper abdomen and pelvis (slice thickness ≤ 10 mm; if contrast CT is not possible due to allergy, non-contrast CT is acceptable)

6) Upper gastrointestinal endoscopy (histopathological examination)

7) Chest X-ray (one direction): lung field condition

8) Resting 12-lead ECG

9) Pulmonary function test: FEV1.0%, %VC

## **Intraoperative and Postoperative Assessment Items**

**8-2-1 Surgical Assessment Items**

1. Surgical procedure, reconstruction method
2. Operative time
3. Blood loss (from incision to closure), transfusion volume (intraoperative and until first discharge)
4. Primary tumor site
5. Tumor size
6. Depth of invasion (post-resection), lymph node metastasis (post-resection), surgical stage (post-resection)
7. Omentectomy
8. Intraoperative complications (CTCAE v3.0 Short Name and JCOG intraoperative/postoperative complication criteria): from incision to closure. The attending physician's judgment regarding causality with the treatment will also be reported.

􀂂 Intraoperative/postoperative complication criteria: pulmonary embolism, lung infarction

􀂂 Surgical/intraoperative injury: pancreas, spleen, gallbladder-common bile duct, portal vein, major arteries of organs, major veins of organs, esophagus, duodenum, jejunum, ileum, colon

􀂂 Other fatal complications

**8-2-2 Postoperative In-Hospital Assessment Items**

1) Early postoperative complications (CTCAE v3.0 Short Name, JCOG intraoperative/postoperative complication criteria, Clavien-Dindo classification) up to 90 days postoperatively.

􀂂 Assessment items: pancreatic fistula, postoperative bleeding, intra-abdominal abscess, gastrointestinal anastomotic leakage, gastrointestinal stricture, cholecystitis, dumping syndrome, delayed gastric emptying, reflux esophagitis, obstructive ileus, paralytic ileus, thrombosis/embolism, postoperative pneumonia, pleural effusion, chylous ascites, surgical site infection, wound dehiscence, other Grade ≥3 non-hematological toxicities

2) Date of first discharge after surgery

**8-2-3 Postoperative Assessment Items**

1) Pathological findings

􀂂 Histological type of primary lesion

􀂂 Depth of invasion, lymph node metastasis

􀂂 Proximal and distal margins

􀂂 Histopathological stage, overall curability

􀂂 Details of each lymph node metastasis

2) Recurrence status

􀂂 Regular follow-up as described below will be conducted to assess recurrence by the attending physician or study office. Recurrence sites will also be recorded.

- 1. Tumor markers (CEA, CA19-9): every 3 months for 3 years postoperatively
  2. CT of upper abdomen and pelvis: every 6 months for 3 years postoperatively
  3. Upper gastrointestinal endoscopy: once a year only for distal gastrectomy

Schedule Table

| Item | Pre-op | Post-op Observation Period | | | | | | | | | | | |
| --- | --- | --- | --- | --- | --- | --- | --- | --- | --- | --- | --- | --- | --- |
| Time | 2–4 weeks before | 3M | 6M | 9M | 1Y | 1Y  3M | 1Y  6M | 1Y  9M | 2Y | 2Y  3M | 2Y  6M | 2Y  9M | 3Y |
| Consent acquisition | ● |  |  |  |  |  |  |  |  |  |  |  |  |
| Patient background | ● |  |  |  |  |  |  |  |  |  |  |  |  |
| Subjective/objective symptoms | ● | ● | ● | ● | ● | ● | ● | ● | ● | ● | ● | ● | ● |
| Adverse events observation ^a^ |  | ● | ● | ● | ● | ● | ● | ● | ● | ● | ● | ● | ● |
| Upper GI endoscopy | ● |  |  |  | ● |  |  |  | ● |  |  |  | ● |
| CT scan | ● |  | ● |  | ● |  | ● |  | ● |  | ● |  | ● |
| Hematology tests ^b^ | ● | ● | ● | ● | ● | ● | ● | ● | ● | ● | ● | ● | ● |
| Biochemistry tests ^c^ | ● | ● | ● | ● | ● | ● | ● | ● | ● | ● | ● | ● | ● |
| Physiological function tests | ● |  |  |  |  |  |  |  |  |  |  |  |  |

M: Month、Y: Year

a: Adverse events include all undesirable events, regardless of causality with the drug.

b: Hematology tests include WBC, hemoglobin, platelet count; used to confirm safety.

c: Biochemistry tests include TP, albumin, AST, ALT, total bilirubin, creatinine, CRP, CEA, CA19-9; used to confirm safety and treatment effect.

# **Criteria for Discontinuation of Individual Subjects**

The principal investigator or sub-investigator will discontinue subjects from the study if continuation becomes difficult for the following reasons, and take appropriate measures based on the subject’s benefit. Investigations/examinations scheduled will be conducted as promptly as possible (except when consent withdrawal prevents execution). If consent is withdrawn after the start of study drug administration, efforts will be made to clarify whether it was due to lack of efficacy, adverse events, or incidental events (e.g., relocation) and record it as reference for inclusion in efficacy/safety evaluation.
 The investigator will record the discontinuation date, reason, and comments in the case report form.

1) Protocol treatment cannot continue due to adverse events

2) Subject requests discontinuation for reasons unrelated to adverse events (e.g., before starting treatment after registration)

3) Death during protocol treatment

4) Other protocol violations

The discontinuation date is defined as the date of death for 3) or the date treatment discontinuation is determined for other cases.

Rationale:

1, 3) To confirm safety

2, 4) Ethical considerations

# **Handling of Adverse Events**

## **Response to Subjects for Adverse Events**

“Adverse event” refers to any undesirable or unintended sign, symptom, or disease occurring after study drug administration, regardless of causality.
 Investigators will take appropriate measures immediately upon recognizing an adverse event and record it accurately in both medical records and CRF. If study drug administration is discontinued or treatment is required for the adverse event, subjects will be informed.
 In emergencies requiring identification of the study drug, the principal investigator will contact the allocation/coding manager through the study representative to obtain disclosure of the allocation for that subject.

## **Reporting of Serious Adverse Events**

　“Serious adverse events” include any undesirable medical event regardless of dose that meets any of the following:

(1) Death

(2) Life-threatening

(3) Requires or prolongs hospitalization

(4) Results in persistent or significant disability/incapacity

(5) Causes congenital anomaly in offspring

Investigators will promptly report all serious adverse events during surgery or postoperative hospitalization to the hospital director (or Clinical Trial Review Board at Osaka City University) regardless of causality with the study. In multicenter trials, if causality cannot be ruled out, the investigator will also report to other institutions’ investigators.

# **Study Completion, Discontinuation, or Interruption**

## **Study Completion**

　Upon completion at each site, the investigator will submit the study completion report to the hospital director. In multicenter trials, a report will also be submitted to the study representative.

## **Discontinuation or Interruption of the Study**

　The principal investigator will review continuation of the study if:

1) Significant information on study drug quality, safety, or efficacy is obtained.

2) Recruitment difficulties make it impossible to achieve the target number of subjects.

3) The study objective is achieved before reaching the planned sample size or period (e.g., via interim analysis).

4) If the Data and Safety Monitoring Committee (DSMC) issues instructions to modify the protocol or other aspects of the trial, and it is judged difficult to comply with such instructions, the trial may be discontinued.

In addition, if the DSMC recommends or instructs termination, the trial will be stopped. For multicenter trials, the principal investigator or the committee specified in the protocol shall review the above matters and determine whether the trial should continue.
 When a decision is made to discontinue or interrupt the trial, the hospital director must be promptly notified in writing with the reasons.

# **Endpoints**

## **Primary Endpoint**

　Relapse-Free Survival (RFS)

【Rationale】

Although the true endpoint to evaluate treatment efficacy is overall survival (OS), this trial is an exploratory phase II study to examine the significance of extensive intraperitoneal lavage for the target cases. It is desirable that results be obtained early. Therefore, RFS, which is an important surrogate endpoint for OS, was set as the primary endpoint.

【Definition of Recurrence】

　Recurrence is defined as follows (definitions of measurable and non-measurable lesions follow RECIST^10^:

1. Detection of any new measurable lesion on postoperative follow-up CT, defined as:
2. Tumor lesion: maximum diameter ≥ 10 mm　② Lymph node lesion: short axis ≥ 15 mm
3. Presence of non-measurable lesions as described below, along with tumor marker levels exceeding the upper limit of normal at the institution.

*Non-measurable lesions:* Small lesions (tumor lesions with long axis < 10 mm or lymph nodes with short axis ≥ 10 mm and < 15 mm), and all lesions other than measurable lesions, including true non-measurable lesions. True non-measurable lesions include leptomeningeal disease, ascites, pleural or pericardial effusion, inflammatory breast cancer, lymphangitic spread in skin or lungs, and abdominal masses or organ enlargement detectable by palpation but not reproducible by imaging.

RFS is defined as the number of days from surgery to either meeting the above criteria for relapse or death.

## **Secondary Endpoints**

1. Overall survival (OS), ② Sites of postoperative recurrence, ③Incidence of postoperative adverse events

【Rationale】

1. OS serves as the true measure of treatment outcome.
2. Recurrence patterns help understand relapse forms in the trial group.
3. Adverse events are important for ensuring trial safety.

# **Data Collection**

## **Case Report Forms (CRF)**

The following CRFs will be used in this trial:

(1) Case registration form

(2) Eligibility confirmation form

(3) Pre-treatment report

(4) Operative findings record

(5) Postoperative records 1 and 2

(6) Pathology report

(7) Progress report

(8) Treatment completion report

## **Document Storage**

Documents related to trial conduct, including (1)-(3) and (5), will be stored by the trial office in lockable cabinets, with keys held by the principal investigator. Operative findings (4) are managed at each participating site. Documents shall be retained until 5 years after publication of research results.

(1) Copies of application documents

(2) Notifications from the ethics committee

(3) Copies of various applications and reports

(4) Consent forms and records of patient consent

(5) CRFs

# **Statistical Analysis**

## **Data Handling**

Data obtained using methods deviating from the protocol will be reviewed with medical experts before trial completion, and responses to unforeseen issues will be decided collaboratively. For protocol-specified observations and tests, data collected outside the allowable time window will be treated as missing. Missing values will not be imputed or estimated.

## **Efficacy Analysis**

Prior to describing the analysis method, the analysis population is defined. Patients registered according to Section 5.1, excluding duplicate or misregistered cases, constitute the "All Registered Patients." Excluding ineligible patients defines the "All Eligible Patients," which is the analysis population.
 The trial treatment will be judged effective if the lower bound of the 90% confidence interval for RFS in all eligible patients exceeds the threshold of 0.86 years.

## **Safety Analysis**

Adverse events (AEs) and adverse reactions (ARs) will be evaluated using the Japanese translation of the Common Terminology Criteria for Adverse Events v4.02 (CTCAE v4.0, MedDRA 12.0/12.1) and Clavien-Dindo classification. Grading is based on definitions closest to Grades 0–4. If the grade description includes specific interventions, grading will reflect clinical necessity (e.g., pleural effusion requiring oxygen or drainage even if patient refuses).
 For AEs specified in Section 8.2, record grade and onset date on the treatment progress CRF. For other AEs, record only Grade ≥3 in the free-text section with AE details and onset date.

# **Trial Period**

Patient enrollment: From approval to March 31, 2019

Patient follow-up: From approval to March 31, 2022

# **Ethical Considerations**

## **Compliance with Guidelines and Declaration of Helsinki**

The trial will be conducted in accordance with the Ethical Guidelines for Medical and Health Research Involving Human Subjects (Dec 22, 2014, MEXT/MHLW) and the Declaration of Helsinki.

## **Ethics Committee Approval**

This study has been approved by the Institutional Review Board of Osaka City University Hospital.。

## **Provision of Explanation Documents and Information to Subjects**

Approved explanatory and consent documents by each facility's review board shall be provided to patients, and sufficient explanation shall be given both in writing and verbally. Written informed consent shall be obtained based on the patient’s free will.
 When information that may affect the patient’s consent, such as efficacy or safety data, is obtained, or when changes to the study protocol that may affect consent are made, patients shall be promptly informed, and their willingness to participate shall be reconfirmed. Explanatory and consent documents shall be revised with prior approval from each facility's review board, and re-consent shall be obtained.

The explanatory documents shall include the following items:

1. Introduction: About the investigator-initiated clinical trial

2. Your medical condition

3. Purpose of this clinical trial

4. Methods of this trial

5. Planned participation period

6. Expected benefits and risks

7. Alternative treatments if you do not participate

8. Treatment costs

9. Possibility of discontinuation of this trial

10. Obligations when you consent to participate

11. Measures if health damage occurs during the trial

12. Intellectual property rights and conflicts of interest arising from the research

13. 　Participation is voluntary (for device trials, include handling of the device if participation is withdrawn)

14. Information regarding this trial will be provided as necessary

15. Privacy will be protected even if trial results are published

16. 　Your medical records may be reviewed during or after the trial

17. Storage, use, and disposal of samples and materials after the study

18. Contact point for inquiries

## **Consideration for Human Rights (Protection of Personal Information)**

During implementation, the rights, welfare, and safety of subjects shall be maximally ensured. The principal investigator or co-investigators shall exercise careful ethical consideration and provide sufficient explanation of the trial, and obtain written consent from the patient. Participation is voluntary and may be withdrawn at any time without disadvantage. Any information obtained in this trial shall not be published in a manner that identifies individuals and shall be strictly protected. If the subject wishes, information will be reported to the subject in writing only.

## **Consideration for Safety and Disadvantages**

Although the likelihood of increased adverse events due to the study treatment is considered low, these data will be collected as secondary endpoints. Monitoring will be conducted twice a year by the study office and the Safety and Efficacy Evaluation Committee to assess whether adverse events fall within the expected range.

If any adverse events occur during participation, the attending physician shall promptly take necessary measures (tests, treatment, trial discontinuation, etc.) to ensure patient safety.

## **Handling of Inquiries**

Appropriate and prompt responses shall be provided to consultations, inquiries, or complaints from subjects, their representatives, or related parties.

# **Study Costs**

## **Study Funding and Conflicts of Interest**

The costs required for this trial are limited to those related to Papanicolaou staining for imprint cytology. These are covered by departmental research funds, research grants, MEXT scientific research grants, and other research subsidies.

## **Patient Cost Burden**

The study treatment will be conducted within the scope of insured medical care. Except for the cost of imprint cytology described in Section 17-1, no non-insured drugs or tests will be performed. The frequency of outpatient visits and tests will be similar to usual care, and the patient’s burden is not expected to increase due to trial participation.

## **Compensation and Insurance for Health Damage**

If health damage arises from participation, treatment will be provided under the patient’s health insurance, as in routine care. No compensation will be provided by the study-related organizations, facilities, or physicians for out-of-pocket expenses, lost income, or extra hospital charges. The attending physician will ensure prompt and appropriate medical care.

# **Record Retention**

The principal investigator shall retain all trial-related documents (copies of applications, notifications from the head of the research institution, copies of various applications and reports, subject identification code list, consent forms, CRFs, and any other documents necessary to ensure data reliability) at the Department of Cancer Molecular Pathophysiology for five years after trial completion.

# **Trial Registration and Publication of Results**

After trial completion and data locking, analyses will be performed for all endpoints. The study office shall promptly compile the results and publish them in appropriate journals and conferences. Final analysis reports will be made after the trial period. When results are published, the hospital director shall also be notified without delay.

# **Study Organization**

## **Principal Investigator**

Masaichi Ohira
Department of Surgical Oncology, Osaka City University Graduate School of Medicine
E-mail: masaichi@med.osaka-cu.ac.jp
1-4-3 Asahimachi, Abeno-ku, Osaka 545-8585, Japan
Tel: +81-6-6645-3838 / Fax: +81-6-6646-6450

## **Participating Facilities & Principal Investigators**

Masaichi Ohira
Department of Surgical Oncology, Osaka City University Graduate School of Medicine
E-mail: masaichi@med.osaka-cu.ac.jp
1-4-3 Asahimachi, Abeno-ku, Osaka 545-8585, Japan
Tel: +81-6-6645-3838 / Fax: +81-6-6646-6450

Naoshi Kubo
Department of Gastroenterological Surgery, Osaka City General Medical Center
E-mail: k-naoshi@med.osaka-cu.ac.jp
06-6929-1221（代）FAX 06-6929-1090

## **Administrative Office**

**Yuichiro Miki, M.D., Ph.D.**
Department of Surgical Oncology / Molecular Oncology and Therapeutics,
Graduate School of Medicine, Osaka City University
1-4-3 Asahi-machi, Abeno-ku, Osaka 545-8585, Japan
Tel: +81-6-6645-3838 Fax: +81-6-6646-6450
E-mail: y_miki@med.osaka-cu.ac.jp

**Masakazu Yashiro, M.D., Ph.D.**Department of Surgical Oncology / Molecular Oncology and Therapeutics,
Graduate School of Medicine, Osaka City University
1-4-3 Asahi-machi, Abeno-ku, Osaka 545-8585, Japan
Tel: +81-6-6645-3838 Fax: +81-6-6646-6450
E-mail: m9312510@med.osaka-cu.ac.jp

## **Safety and Efficacy Evaluation Committee**

**Tetsuji Sawada, M.D., Ph.D.**

Director, Osaka Ekisaikai Hospital

E-mail: [t.sawada@osaka-ekisaikai.jp](mailto:t.sawada@osaka-ekisaikai.jp)

**Tasuku Matsuoka, M.D., Ph.D.**

Chief of Surgery, Naniwa Ikuno Hospital

E-mail: tsubasam1965@yahoo.co.jp

## **Monitoring Officer**

**Hisashi Nagahara, M.D., Ph.D.**

Department of Surgical Oncology, Osaka City University Graduate School of Medicine

1-4-3 Asahi-machi, Abeno-ku, Osaka 545-8585, Japan
Tel: +81-6-6645-3838 Fax: +81-6-6646-6450

E-mail: hisashi@med.osaka-cu.ac.jp

## **Biostatistician**

**Mitsuru Fukui**

Department of Urban Health, Osaka City University Graduate School of Medicine

1-4-3 Asahi-machi, Abeno-ku, Osaka 545-8585, Japan
Tel: +81-6-6645-3838

E-mail: fukui@med.osaka-cu.ac.jp

# **Protocol Deviations or Amendments**

Any revisions to the protocol or explanatory/consent documents require prior approval from the Clinical Trial / Ethics Committee. Exceptions include changes urgently needed to prevent medical risk. Major deviations must be recorded with reasons. If important information regarding trial drug quality, efficacy, or safety is obtained, protocol amendments may be made and major deviations documented with reasons.

# **Monitoring / Data Quality Management**

On-site monitoring may be conducted to improve scientific and ethical quality and for educational purposes. Monitors verify that the trial is properly conducted, necessary items are accurately recorded, and data reliability is maintained.

# **Audits**

On-site audits may be conducted to improve scientific and ethical quality and for educational purposes. Facility selection will be conducted according to the audit procedures separately established.

# **References**

**1.** Health Statistics Association. Trends in Public Health. Vol 42.

**2.** Sakuramoto S, Sasako M, Yamaguchi T, et al. Adjuvant chemotherapy for gastric cancer with S-1, an oral fluoropyrimidine. *N Engl J Med.* Nov 1 2007;357(18):1810-1820.

**3.** Noh SH, Park SR, Yang HK, et al. Adjuvant capecitabine plus oxaliplatin for gastric cancer after D2 gastrectomy (CLASSIC): 5-year follow-up of an open-label, randomised phase 3 trial. *Lancet Oncol.* Nov 2014;15(12):1389-1396.

**4.** Sasako M, Sakuramoto S, Katai H, et al. Five-year outcomes of a randomized phase III trial comparing adjuvant chemotherapy with S-1 versus surgery alone in stage II or III gastric cancer. *J Clin Oncol.* Nov 20 2011;29(33):4387-4393.

**5.** Bando E, Yonemura Y, Takeshita Y, et al. Intraoperative lavage for cytological examination in 1,297 patients with gastric carcinoma. *American journal of surgery.* Sep 1999;178(3):256-262.

**6.** Kodera Y, Imano M, Yoshikawa T, et al. A randomized phase II trial to test the efficacy of intra-peritoneal paclitaxel for gastric cancer with high risk for the peritoneal metastasis (INPACT trial). *Jpn J Clin Oncol.* Feb 2011;41(2):283-286.

**7.** Kuramoto M, Shimada S, Ikeshima S, et al. Extensive intraoperative peritoneal lavage as a standard prophylactic strategy for peritoneal recurrence in patients with gastric carcinoma. *Ann Surg.* Aug 2009;250(2):242-246.

**8.** Masuda T, Kuramoto M, Shimada S, et al. The effect of extensive intraoperative peritoneal lavage therapy (EIPL) on stage III B + C and cytology-positive gastric cancer patients. *International journal of clinical oncology.* Apr 2016;21(2):289-294.

**9.** Fujitani K, Yang HK, Kurokawa Y, et al. Randomized controlled trial comparing gastrectomy plus chemotherapy with chemotherapy alone in advanced gastric cancer with a single non-curable factor: Japan Clinical Oncology Group Study JCOG 0705 and Korea Gastric Cancer Association Study KGCA01. *Jpn J Clin Oncol.* Jul 2008;38(7):504-506.

**10.** Eisenhauer EA, Therasse P, Bogaerts J, et al. New response evaluation criteria in solid tumours: revised RECIST guideline (version 1.1). *European journal of cancer (Oxford, England : 1990).* Jan 2009;45(2):228-247.
