# Supplementary material for: Extensive intraoperative peritoneal lavage (EIPL) for gastric cancer with positive peritoneal lavage and/or stamp cytology: An exploratory phase II study
Source: PLoS One. 2026 Apr 17;21(4):e0347742. doi: 10.1371/journal.pone.0347742 (PMC13089869; doi:10.1371/journal.pone.0347742)
Supplement: S3 File — (DOCX) [file pone.0347742.s003.docx]

**胃壁捺印細胞診または腹腔洗浄細胞診陽性症例を対象とした**

**術中腹腔内大量洗浄の意義に関する第Ⅱ相試験**

A phase II study to **e**valuate the intraoperative peritoneal lavage treatment for patients who are diagnosed as positive by peritoneal lavage cytology or stamp cytology

**研究実施計画書**

研究代表者

大平雅一

大阪市立大学大学院医学研究科　腫瘍外科学講座

E-mail： masaichi@med.osaka-cu.ac.jp

〒545-8585  大阪市阿倍野区旭町1-4-3

 06-6645-3838（代）FAX 06-6646-6450

事務局

三木友一朗

大阪市立大学大学院医学研究科　腫瘍外科学、癌分子病態制御学

E-mail： y_miki@ med.osaka-cu.ac.jp

〒545-8585  大阪市阿倍野区旭町1-4-3

06-6645-3838（代）FAX 06-6646-6450

八代正和

大阪市立大学大学院医学研究科　腫瘍外科学、癌分子病態制御学

E-mail： m9312510@med.osaka-cu.ac.jp

〒545-8585  大阪市阿倍野区旭町1-4-3

06-6645-3838（代）FAX 06-6646-6450

初版：2016年12月27日

大阪市立大学医学研究科　臨床試験・治験審査委員会承認

2016年12月28日

（承認番号203664）

# **概要**

## **シェーマ**

**進行胃癌に対してD2リンパ節郭清を伴う胃切除術を予定する症例**

・20歳以上80歳以下　・PS 0/1

**術前同意取得**

試験への登録

陰性症例は不適格

術中、腹腔洗浄細診および捺印細胞診施行

いずれかに陽性症例を適格症例とする。

手術中、閉腹前に12Lの生理食塩水で腹腔内洗浄

追跡期間：術後3年間

<<B群

手術中、閉腹前に2Lの生理食塩水で腹腔内洗浄

## **背景**

- 根治切除された胃癌症例においても腹膜再発は多い。
- 腹膜再発の予測にこれまで用いられてきた方法に腹腔洗浄細胞診があるが、感度の低さが問題としてあった。当院での過去の検討では捺印細胞診を付加的に行うことで感度の上昇に寄与する事が示されている。
- 通常、胃切除後は2L程度の生理食塩水で腹腔内を洗浄することが一般的であるが、腹腔洗浄細胞診陽性症例においては腹腔内を大量に生食で洗浄することで生存転帰の向上をもたらすとの報告がある。
- しかしながら、腹腔洗浄細胞診または捺印細胞診陽性症例を対象として、腹腔内大量洗浄を行うことの無再発生存期間への影響を前向きに検討した報告は過去にない。

## **目的**

　D2リンパ節郭清を伴う胃切除術を予定している症例において、術中腹腔洗浄細胞診または胃壁捺印細胞診陽性症例を対象として、腹腔内大量洗浄による無再発生存期間における優越性を非対照の第II相試験にて検討する。

　また安全性に関する事項もSecondary endpointとしてデータ収集を行う。

Primary endpoint: 無再発生存期間

Secondary endpoints: ① 全生存率、② 術後再発部位、③術後有害事象発生割合

## **対象**

1) 胃原発巣からの内視鏡生検にて、組織学的に胃癌(pap, tub1, tub2, por1, por2, sig, muc, 特殊型のいずれか)と診断されている。

2) 腹腔洗浄細胞診もしくは胃壁捺印細胞診陽性であるが、その他に遠隔転移を認めず、D2リンパ節郭清を伴う胃切除術（胃全摘術もしくは幽門側胃切除術、腹腔鏡手術を含む）によりR0/1切除の完遂が可能である。その際の麻酔方法は全身麻酔、硬膜外麻酔の併用で行う。

3) 食道浸潤が3cm以内であり、かつ開胸操作が行われていない。

4) 20歳以上80歳以下である。

5) PS (ECOG)が0または1である。

6) 他のがん腫に対する治療も含めて化学療法（内分泌療法を含む）や放射線治療の既往がない。

7) 下記のすべての条件をみたす。（すべての検査項目は登録前56日以内の最新の術前検査値を用いる。登録日の8週間前の同じ曜日の検査は許容する。

①　白血球数: 3,000/mm^3^以上1,0000/mm^3^以下

②　血小板数≧100,000/mm^3^

③　AST≦100 IU/L、ALT≦100IU/L

④　総ビリルビン≦2.0 g/dl

⑤　血清クレアチニン≦1.5mg/dl

8) 試験参加について、患者本人から文書で同意が得られている。

## **治療**

胃癌治療ガイドライン（医師用第3版）で規定される、D2以上のリンパ節郭清を伴う手術を行う。

手術中、閉腹前に腹腔内を12Lの生理食塩水により洗浄を行う。

## **予定適格症例数と研究期間**

予定適格症例数：　65名

登録期間：　2年、追跡期間：登録終了後3年、総研究期間：　5年

## **問い合わせ先**

研究事務局

三木友一朗

大阪市立大学大学院医学研究科　腫瘍外科学、癌分子病態制御学

E-mail： y_miki@ med.osaka-cu.ac.jp

〒545-8585  大阪市阿倍野区旭町1-4-3

06-6645-3838（代）FAX 06-6646-6450

八代正和

大阪市立大学大学院医学研究科　腫瘍外科学、癌分子病態制御学

E-mail： m9312510@med.osaka-cu.ac.jp

〒545-8585  大阪市阿倍野区旭町1-4-3

 06-6645-3838（代）FAX 06-6646-6450

**目次**

**0** **概要** i

**シェーマ** i

**0.1** **背景** i

**0.2** **目的** i

**0.3** **対象** ii

**0.4** **治療** ii

**0.5** **予定適格症例数と研究期間** ii

**0.6** **問い合わせ先** iii

1 **試験の背景** 1

**1.1** **本試験の意義** 2

**1.2** **付随研究** 2

**2** **試験薬の概要** 2

**2.1** **試験薬** 2

**2.2** **予期される有害反応および医療機器の場合は予期される不具合** 2

**3** **対象疾患の診断基準と病期・病型分類** 2

**3.1** **胃癌取扱い規約および胃癌治療ガイドライン** 2

**3.2** **Performance Status（PS）の評価** 2

**4** **対象患者** 3

**4.1** **選択基準（組み入れ基準）** 3

**4.2** **除外基準^9^** 3

**5** **症例登録** 4

**5.1** **登録の手順** 4

**6** **試験計画** 4

**6.1** **試験の種類・デザイン** 4

**6.2** **臨床的仮説と登録数設定根拠** 5

**6.3** **登録集積見込み** 5

**6.4** **試験のアウトライン** 6

**6.5** **被験者の試験参加期間** 6

**6.6** **試験薬の用法・用量、投与期間** 6

**6.7** **併用薬（療法）に関する規定** 6

**6.8** **試験薬の管理・交付手順** 6

**6.9** **試験終了後の対応（または「後治療」）** 6

**6.10** **試料等の保存および他の機関等の試料等の利用** 6

**7** **観察および検査項目とスケジュール** 7

**7.1** **8-1　登録前評価項目** 7

**7.2** **術中・術後の評価項目** 7

**8** **個々の被験者の中止基準** 9

**9** **有害事象発生時の取扱い** 9

**9.1** **有害事象発生時の被験者への対応** 9

**9.2** **重篤な有害事象の報告** 10

**10** **試験の終了、中止、中断** 10

**10.1** **試験の終了** 10

**10.2** **試験の中止、中断** 10

**11** **評価項目** 10

**11.1** **主要評価項目** 10

**11.2** **副次的評価項目** 11

**12** **データの集計** 11

**12.1** **記録用紙（Case report form: CRF）** 11

**12.2** **記録の保管** 11

**13** **統計解析** 12

**13.1** **データの取り扱い** 12

**13.2** **有効性解析** 12

**13.3** **安全性解析** 12

**14** **試験実施期間** 13

**15** **倫理的事項** 13

**15.1** **指針及びヘルシンキ宣言への対応** 13

**15.2** **倫理委員会の承認** 13

**15.3** **同意・説明文書及び被験者への情報提供** 13

**15.4** **人権への配慮（個人情報の保護）** 14

**15.5** **安全性・不利益への配慮** 14

**15.6** **相談等の対応** 14

**16** **試験の費用負担** 14

**16.1** **試験資金および利益相反** 14

**16.2** **患者の費用負担** 14

**16.3** **健康被害の補償および保険への加入** 14

**17** **記録の保存** 15

**18** **試験計画の登録および試験結果の公表** 15

**19** **試験組織** 15

**研究代表者** 15

**19.1** **実施施設・試験責任医師** 15

**19.2** **試験事務局** 15

**19.3** **効果安全性評価委員会** 16

**19.4** **モニタリング担当者（モニター）** 16

**19.5** **生物統計家** 16

**20** **実施計画書からの逸脱または変更** 16

**21** **モニタリング／データの品質管理** 17

**22** **監査** 17

**23** **参考文献** 18

# **試験の背景**

わが国における胃癌の死亡率は、男女とも1970年代から低下傾向が続き、男性では1993年以降肺癌がこれを上回ったが、悪性腫瘍による死亡原因の第2位である^1^。

胃癌治療ガイドライン（第3版）によればN(+)もしくはT2以深の腫瘍に対する定型手術は幽門側胃切除術もしくは胃全摘術であると述べられている。幽門側胃切除術により適切な切離断端を確保出来ない場合が胃全摘術の適応となる。進行胃癌に対するリンパ節郭清については本邦で行われたJCOG9501試験の結果を受けて、D2郭清が標準治療である。治癒切除後の補助療法については、ACTS-GC試験（Adjuvant Chemotherapy Trial of TS-1 for Gastric Cancer）の結果をうけて、pStage II～IIIB（pT1 を除く）の胃癌患者に対しては術後にTS-1 を1 年間内服することが我が国の標準治療となった[^2^](#_ENREF_2)。さらに手術単独とカペシタビン、オキサリプラチンの併用 (CapeOX)による補助化学療法を比較したランダム化比較第三相試験 (CLASSIC試験)の結果を受け、CapeOX療法も術後補助化学療法として「推奨される治療」とされている[^3^](#_ENREF_3)。

　これらの治療開発により胃癌手術症例の生存成績は向上してきている。しかしながら、根治切除された胃癌症例でも腹膜転移再発は37-39.4%との報告もある(JCOG9206-1、JCOG9206-2, JCOG9501試験)。またいずれの試験においても胃癌に対する根治切除後の再発部位としては腹膜転移が最多であった。先述のACTS-GC試験では補助化学療法施行症例においても14.6% (77/549)において腹膜転移再発が見られた[^4^](#_ENREF_4)。これらの結果は現在の病期診断において根治切除と判定されている症例の中にも腹膜播種再発の高リスク症例が含まれている事を意味している。

　腹膜播種再発の予測に現時点では腹腔洗浄細胞診 (CY)が最も広く用いられている。CY陽性症例の5年全生存率は0-2%程度と報告されており、その生存転帰は極めて不良である事から、この対象に対する治療成績向上には新規の治療開発が望まれる[^5^](#_ENREF_5)^,^[^6^](#_ENREF_6)。

　しかしながら現在のCYの問題点として再発予測における感度の低さが挙げられる。当施設で過去に行った試験において、捺印細胞診を通常のCY と併用する事で感度が25.0%から47.1%に上昇する事が示されている。腹膜再発の可能性が高い症例をできるだけ多く治療の対象に含めた新規の治療開発が重要と考え、捺印細胞診陽性症例に対しても新規の治療開発を進める必要があると考える。

過去には、CY陽性症例を対象として、胃切除に加えて腹腔内大量洗浄および腹腔内化学療法の意義を検討する小規模なRCTが行なわれている[^7^](#_ENREF_7)。その中では「腹腔内大量洗浄、腹腔内化学療法の併用」が最も治療成績が良好であったが、「腹腔内大量洗浄単独」においても「手術単独」群よりも治療成績は良好であった。またsingle armの試験ではあるものの、腹腔内大量洗浄によりCY1P0症例における5年生存率46.5%という成績も報告されており、腹腔内大量洗浄単独でも再発予防に寄与する可能性があると考える[^8^](#_ENREF_8)。しかしながら、CY陽性または捺印細胞診陽性症例を対象に腹腔内大量洗浄の生存に対する効果を前向きに検討した試験はこれまでに存在しない。

先述の過去の報告では10Lの腹腔内洗浄を行っている。我々は通常、手術終了時に2Lの洗浄を行っているため、通常の方法に加えて10L洗浄を行う12Lの洗浄の効果を評価する目的で本試験における試験治療として設定した。また手術終了直前に腹腔内大量洗浄を行う事で重篤な有害事象の出現は考えにくく、本試験治療は施設を問わずに簡便に行う事が可能な治療である。これらより、今回、CY陽性または捺印細胞診陽性症例を対象に12Lの腹腔内洗浄による無再発生存期間における優越性を非対照の第II相試験にて検討する事とした。

## **本試験の意義**

進行胃癌に対する標準治療はD2郭清を伴う胃切除術および術後補助化学療法であるが、術後再発の頻度は十分には低くなく、さらなる治療開発が望まれる。特に頻度の高い腹膜再発の克服は重要な課題であり、本試験において腹腔内大量洗浄の有効性を評価する事は意義があると考える。

## **付随研究**

本試験では、腹水洗浄細胞診陽性症例と捺印細胞診陽性症例のサブグループ解析を計画している。また術中出血量、輸血量が再発に関連があるとの報告があることから、それらに関するデータも追加で検討する予定である。術中に胃壁を擦過した検体を採取し、擦過細胞の遺伝子発現に関する情報と臨床病理学的因子、予後との関連も検討する事を予定している。

今後、本試験のデータを用いた付随研究を新たに提案する場合は、検討したい内容を文書にまとめ、本研究代表者に提出する。研究代表者は提案のあった付随研究の内容と実施方法について提案者とともに検討する。

# **試験薬の概要**

## **試験薬**

生理食塩水 （ナトリウムイオン 154mEq/L, 塩素イオン154mEq/L）を用いる。

## **予期される有害反応および医療機器の場合は予期される不具合**

上記試験薬を用いた腹腔内の洗浄で予期される有害事象は過去の報告上ない。大量に静脈内に投与すると、血清電解質異常、うっ血性心不全、浮腫、アシドーシスを起こすことがある。

# **対象疾患の診断基準と病期・病型分類**

## **胃癌取扱い規約および胃癌治療ガイドライン**

本プロトコールでの表記は、胃癌取扱い規約第14版および胃癌治療ガイドライン第3版に従う。

## **Performance Status（PS）の評価**

ECOG scaleの日本語訳を用いる。

| Grade | | Performance Status |
| --- | --- | --- |
| 0 | 無症状で社会活動ができ，制限を受けることなく発病前と同等にふるまえる。 | |
| 1 | 軽度の症状があり，肉体労働は制限を受けるが，歩行，軽労働や坐業はできる。  例えば軽い家事，事務など。 | |
| 2 | 歩行や身の回りのことはできるが，時に少し介助がいることもある。  軽労働はできないが，日中の50%以上は起居している。 | |
| 3 | 身の回りのある程度のことはできるが，しばしば介助がいり，日中の50%以上は就床している。 | |
| 4 | 身の回りのこともできず，常に介助がいり，終日就床を必要としている。 | |

# **対象患者**

以下の選択規準をすべて満たし、除外規準のいずれにも該当しない患者を登録適格患者とする。病期分類、組織型分類などは胃癌取扱い規約第14 版に従う。

## **選択基準（組み入れ基準）**

1) 胃原発巣からの内視鏡生検にて、組織学的に胃癌(pap, tub1, tub2, por1, por2, sig, muc, 特殊型のいずれか)と診断されている。

2) 腹水洗浄細胞診もしくは胃壁捺印細胞診陽性であるが、その他に遠隔転移を認めず、D2リンパ節郭清を伴う胃切除術（胃全摘術もしくは幽門側胃切除術、腹腔鏡手術を含む）によりR0/1切除の完遂が可能である。その際の麻酔方法は全身麻酔、硬膜外麻酔の併用で行う。

3) 食道浸潤が3cm以内であり、かつ開胸操作が行われていない。

4) 20歳以上80歳以下である。

5) PS (ECOG)が0または1である。

6) 他のがん腫に対する治療も含めて化学療法（内分泌療法を含む）や放射線治療の既往がない。

7) 下記のすべての条件をみたす。（すべての検査項目は登録前56日以内の最新の術前検査値を用いる。登録日の8週間前の同じ曜日の検査は許容する。

①　白血球数: 3,000/mm^3^以上1,0000/mm^3^以下

②　血小板数≧100,000/mm^3^

③　AST≦100 IU/L、ALT≦100IU/L

④　総ビリルビン≦2.0 g/dl

⑤　血清クレアチニン≦1.5mg/dl

8) 試験参加について、患者本人から文書で同意が得られている。

［設定根拠］

１）胃癌と組織学的に証明されている症例である必要があるため

２）腹水洗浄細胞診陽性もしくは捺印細胞診陽性症例は腹膜転移再発の高リスク症例と考えられるため対象とする。しかしながら他の遠隔転移を有する症例はR0/1切除が困難な事が多く、今回の対象から除外した。

3)食道浸潤が3cm以上の症例では開胸が必要となる事があり、今回の対象から除外した。

4）〜7） 特殊な症例、術前の全身状態の悪い症例は除外するために設定した。

8) 患者からの同意は必須と考えられるため設定した。

## **除外基準^[9](#_ENREF_9)^**

1) 妊娠の可能性がある、または授乳中の女性。

2) 精神病または精神症状を合併しており、試験への参加が困難と判断される。

3) ステロイド剤の継続的な全身投与（内服または静脈内）を受けている。

4) 6か月以内の心筋梗塞の既往もしくは不安定狭心症を有している。

5) コントロール不良の高血圧症を合併している。

6) インスリンによる治療中、またはコントロール不良の糖尿病を合併している。

7) 持続酸素投与を要する呼吸器疾患を合併している。

［設定根拠］

１）〜7) 有効性評価への影響および安全性への配慮のため。過去の胃癌症例における臨床試験に準じて設定した。[^9^](#_ENREF_9)

# **症例登録**

## **登録の手順**

登録対象：術前に試験責任医師あるいは試験分担医師は文書による同意を取得する。選択規準（２を除く）を満たし、除外規準のいずれにも該当しない場合、症例登録書に必要事項を記載し試験への登録を行う。

解析対象：手術中に対象患者が選択規準を満たした場合、適格性確認票を記載の上、プロトコール治療を行う。症例登録書、適格性確認票は事務局にて保管する。同意撤回、中止、脱落等が生じた時は、速やかに報告する。

研究事務局

三木友一朗

大阪市立大学大学院医学研究科　腫瘍外科学、癌分子病態制御学

E-mail： y_miki@ med.osaka-cu.ac.jp

〒545-8585  大阪市阿倍野区旭町1-4-3

06-6645-3838（代）FAX 06-6646-6450

八代正和

大阪市立大学大学院医学研究科　腫瘍外科学、癌分子病態制御学

E-mail： m9312510@med.osaka-cu.ac.jp

〒545-8585  大阪市阿倍野区旭町1-4-3

06-6645-3838（代）FAX 06-6646-6450

# **試験計画**

## **試験の種類・デザイン**

本試験はD2リンパ節郭清を伴う胃切除術を予定している症例において、術中腹腔洗浄細胞診または胃壁捺印細胞診陽性症例を対象として、腹腔内大量洗浄による無再発生存期間における優越性を検討する非対照の第II相試験である。登録症例数の設定に際しては、1997年から2012 年において当教室で手術を施行した症例のうち、本試験に適格となる138例における生存転帰を閾値の設定に用いた。

設定根拠：本試験の対象症例は胃癌患者全体のなかでも頻度が低く、症例集積に時間を要する可能性がある。その点においてランダム化比較試験を行う事は困難と考え、過去の症例における治療成績を閾値の設定に用いた単群における第二相試験として計画した。

## **臨床的仮説と登録数設定根拠**

1. **臨床的仮説**

胃癌に対してD2リンパ節郭清を伴う胃切除術を施行した症例のうち術中腹水洗浄細胞診または胃壁捺印細胞診陽性症例を対象として、腹腔内大量洗浄を施行する事で無再発生存期間を延長させうる。

1. **登録数設定根拠**

　1997年から2012 年に当院に手術施行症例のうちCY陽性もしくは捺印細胞診陽性であった症例の無再発生存期間中央値は315日 (0.86年)であった。この値を閾値に設定し、これが今回の試験治療において438日　(1.2年)に延長する事を期待値として設定した。本試験では、α=0.05、β=0.20とすると62例が必要となる。本試験では予定登録数を65例とした。

## **登録集積見込み**

2011年に大阪市立大学医学部付属病院で本試験の登録適格基準を満たす症例は20例であった。同意取得割合を75%と仮定すると、年間登録15例と予測される。単施設での登録では症例集積を短期間に終了する事が困難であり、本試験は多施設共同試験として行う予定である。年間5-15例程度の登録が見込まれる施設施設の参加により年間登録見込みは35例程度となる事から登録期間は2年間とする。また登録終了後3年間の追跡期間が必要であるため総研究期間は5年とする。

## **試験のアウトライン**

| 同意取得 | ⇨ | 登　録 | ⇨ | 手術開始時に検査 | ⇨ | 適格性確認 |  |  |  | 術後3年間のフォロー  採血 (/3ヶ月)  CT (/6ヶ月)  上部消化管内視鏡 (/年) |
| --- | --- | --- | --- | --- | --- | --- | --- | --- | --- | --- |
|  |  |  |  |  |  |  | ⇨ |  |  |  |
|  |  |  |  |  |  |  |  | 手術終了時に  腹腔内大量洗浄施行 |  |  |
|  |  |  |  |  |  |  | ⇨ |  |  |  |
|  |  |  |  |  |  |  |  |  |  |  |

## **被験者の試験参加期間**

全症例術後36ヶ月追跡まで

術後3ヶ月に1回の来院にてフォローを行う（最大12回/36ヶ月）

## **試験薬の用法・用量、投与期間**

　生理食塩水、12Lを用いて手術終了時に腹腔内大量洗浄を行う。

## **併用薬（療法）に関する規定**

１）併用薬（療法）：該当なし。

２）併用禁止薬（療法）：該当なし。

## **試験薬の管理・交付手順**

試験で使用する薬剤は手術部に常備されている薬剤を使用する。

## **試験終了後の対応（または「後治療」）**

被験者が試験終了後においても試験の結果により得られた最善の予防、診断および治療を受けることができるよう努める。

## **試料等の保存および他の機関等の試料等の利用**

　試験に関して得られる捺印細胞診に関する試料については、癌分子病態制御学講座において、試験終了報告後5年間保存する。その際の管理責任に関しては試験事務局で担当する。また管理状況について病院長に報告する。試料の廃棄の際は匿名化された状態で行う。

　研究者等が情報等を正確なものにするよう指導・管理し、人体から取得された試料及び情報等についても試験終了後5年間は保存することとし、漏えい、混交、盗難、紛失等が起こらないよう必要な管理を行う

# **観察および検査項目とスケジュール**

## **8-1　登録前評価項目**

**登録前56日以内に行う検査**

1) 全身状態：PS（ECOG）、身長、体重

2) 末梢血算：白血球数、ヘモグロビン、血小板

3) 血液生化学：AST、ALT、総ビリルビン、クレアチニン、CRP

4) 腫瘍マーカー：CEA、CA19-9

5) 上腹部・骨盤造影CT（スライス幅10 mm 以下、造影剤アレルギーが原因で造影CTが　不可能な場合は単純CTでも可）

6) 上部消化管内視鏡検査（病理組織学的検査）

7) 胸部 X-P（1方向）：肺野条件

8) 安静時 12 誘導心電図

9) 呼吸機能検査：FEV1.0%、%VC

## **術中・術後の評価項目**

**8-2-1 手術の評価項目**

1. 術式、再建方法
2. 手術時間
3. 出血量（開腹より閉腹までのカウント）、輸血量（術中および初回退院まで）
4. 腫瘍主占居部位
5. 腫瘍径
6. 壁深達度（摘出後）、リンパ節転移（摘出後）、手術的進行度（摘出後）
7. 網嚢切除の有無
8. 術中合併症（CTCAE v3.0 Short Name およびJCOG術中・術後合併症規準）： 開腹から手術終了（閉腹）まで。治療との因果関係に関する担当医の判断も報告する。

􀂂 術中・術後合併症規準：肺梗塞・肺塞栓症

􀂂 手術/術中損傷：術中損傷-[膵、脾臓、胆管-総胆管、門脈、臓器の主要な動脈、臓器の主要な静脈、食道、十二指腸、空腸、回腸、結腸]

􀂂 その他の致命的な合併症

**8-2-2 手術後入院中の評価項目**

1) 術後早期合併症（CTCAE v3.0 Short Name、JCOG 術中・術後合併症規準および**Clavien-Dindo分類**）： 術後90日まで。

􀂂 評価項目：膵液瘻、術後出血、腹腔内膿瘍、消化管縫合不全、消化管吻合部狭窄、胆嚢炎、ダンピング症候群、胃排出遅延、逆流性食道炎、閉塞性イレウス、麻痺性イレウス、血栓症/塞栓症、術後肺炎、術後胸水、乳び腹水、術後創感染、創し開、その他のGrade3以上の非血液毒性

2) 術後初回退院日

**8-2-3 手術後の評価項目**

1) 病理所見

􀂂 原発巣の組織型

􀂂 壁深達度、リンパ節転移

􀂂 近位断端、遠位断端

􀂂 組織学的進行度、総合的根治度

􀂂 各リンパ節転移の詳細) 病理所見

2) 再発の有無

􀂂 術後下記に示す定期的なフォローを行い、再発の有無について担当医あるいは事務局が評価する。再発部位についても記録する。

- - 1. 腫瘍マーカー（CEA、CA19-9）：術後3 年間、3 か月に1 回
    2. 上腹部・骨盤造影CT：術後3 年間、6 か月に1 回
    3. 上部消化管内視鏡検査：幽門側胃切除術の場合のみ1 年に1 回

スケジュール表

| 項　目 | 術前 | 後観察期間 | | | | | | | | | | | |
| --- | --- | --- | --- | --- | --- | --- | --- | --- | --- | --- | --- | --- | --- |
| 時　期 | ２～４  週前 | 術後  3M | 術後  6M | 術後  9M | 術後  1Y | 術後  1Y3M | 術後  1Y6M | 術後  1Y9M | 術後  2Y | 術後  2Y3M | 術後  2Y6M | 術後  2Y9M | 術後  3Y |
| 同意取得 | ● |  |  |  |  |  |  |  |  |  |  |  |  |
| 患者背景の確認 | ● |  |  |  |  |  |  |  |  |  |  |  |  |
| 自覚症状  他覚所見 | ● | ● | ● | ● | ● | ● | ● | ● | ● | ● | ● | ● | ● |
| 有害事象の観察^a^ |  | ● | ● | ● | ● | ● | ● | ● | ● | ● | ● | ● | ● |
| 上部消化管  内視鏡検査 | ● |  |  |  | ● |  |  |  | ● |  |  |  | ● |
| CT検査 | ● |  | ● |  | ● |  | ● |  | ● |  | ● |  | ● |
| 血液学的検査^b^ | ● | ● | ● | ● | ● | ● | ● | ● | ● | ● | ● | ● | ● |
| 血液生化学検査^c^ | ● | ● | ● | ● | ● | ● | ● | ● | ● | ● | ● | ● | ● |
| 生理機能検査 | ● |  |  |  |  |  |  |  |  |  |  |  |  |

M: 月、Y: 年

a: 有害事象は、副作用など好ましくないすべての事象のことで、薬との因果関係は問いません。

b: 血液学的検査として白血球数、ヘモグロビン濃度、血小板数を測定。これらは試験の安全性を確認を目的とする。

c: 血液生化学検査としてTP、アルブミン、AST、ALT、総ビリルビン、クレアチニン、CRP 、CEA, CA19-9を測定。これらは試験の安全性確認および試験治療の効果を確認する事を目的とする。

# **個々の被験者の中止基準**

　試験責任医師または試験分担医師は、以下の理由によって試験の継続が困難となった被験者について試験を中止し、被験者の利益性に基づき適切に処置する。また、予定されている調査／検査を可能な限り速やかに実施し、判定を行う。（同意撤回などによって実施できない場合を除く。）試験薬投与開始後に同意の撤回があった場合は、試験薬の効果不発揮あるいは有害事象によるものか、あるいは偶発的事象（転居など）によるものかをできるだけ明らかにし、有効性・安全性評価の対象となる症例としての採否の参考となるように記録する。

　試験責任医師または試験分担医師は、中止日、中止理由、コメントを症例報告書に記録する。

1) 有害事象によりプロトコール治療が継続できない場合

2) 有害事象との関連が否定できる理由により、患者がプロトコール治療の中止を申し出た場合（登録後治療開始前に、患者がプロトコール治療の中止を申し出た場合など）

3) プロトコール治療中の死亡

4) その他、プロトコール違反が判明した場合など

プロトコール治療中止日は、3）の場合は死亡日、それ以外の場合はプロトコール治療中止と判断した日とする。

設定根拠：

1), 3) 安全性の確認のため

2), 4) 倫理的な配慮のため

# **有害事象発生時の取扱い**

## **有害事象発生時の被験者への対応**

　「有害事象」とは、試験薬が投与された時に起こるあらゆる好ましくない、あるいは意図しない徴候、症状または病気とし、試験薬との因果関係の有無は問わない。

試験責任医師または試験分担医師は、有害事象を認めたときは、直ちに適切な処置を行うとともに、カルテならびに症例報告書に齟齬なく記載する。また、試験薬の投与を中止した場合や、有害事象に対する治療が必要となった場合には、被験者にその旨を伝える。

　緊急避難的に試験薬の識別を行う必要がある場合は、研究代表者等を通じて試験薬割付・コード化担当者に依頼し、当該症例について開封結果の開示を受ける。

## **重篤な有害事象の報告**

　「重篤な有害事象」とは、投与量に関わらず、あらゆる好ましくない医療上の出来事のうち、以下の条件に該当するものをいう。

（１）死に至るもの

（２）生命を脅かすもの

（３）治療のため入院または入院期間の延長が必要となるもの

（４）永続的又は顕著な障害・機能不全に陥るもの

（５）子孫に先天異常を来すもの

試験責任医師は、手術中もしくは術後入院期間中に重篤な有害事象の発生を認めたときは、当該研究との因果関係の有無にかかわらず、全ての重篤な有害事象を速やかに病院長 (大阪市立大学においては臨床試験・治験審査委員会)に報告するとともに、多施設共同試験において試験と重篤な有害事象の因果関係を否定できない場合は、他の医療機関の責任医師に報告する。

# **試験の終了、中止、中断**

## **試験の終了**

　各施設での試験の終了時には、試験責任医師は、速やかに試験終了報告書を病院長に提出する。多施設において実施する場合は、研究代表者にも終了報告書を提出する。

## **試験の中止、中断**

　試験責任医師は、以下の事項に該当する場合は試験実施継続の可否を検討する。

１）試験薬の品質、安全性、有効性に関する重大な情報が得られたとき。

２）被験者のリクルートが困難で予定症例を達成することが到底困難であると判断されたとき。

３）予定症例数または予定期間に達する前に（中間解析等により）試験の目的が達成されたとき。

４）効果安全性評価委員会により、実施計画等の変更の指示があり、これを受入れることが困難と判断されたとき。

なお、効果安全性評価委員会により、中止の勧告あるいは指示があった場合は、試験を中止する。多施設で行う場合は、研究代表者あるいは試験計画書で規定する委員会等で上記の事項を検討し、試験の継続の可否を検討する。

試験の中止または中断を決定した時は、速やかに病院長にその理由とともに文書で報告する。

# **評価項目**

## **主要評価項目**

　無再発生存期間

【設定根拠】

　治療の効果を検証する真のendpointは全生存期間であるが、本試験は今回の対象症例に対する腹腔内大量洗浄の意義を検討する探索的なphase II試験であり、結果が早期に判明する事が望まれる。したがって全生存期間のsurrogate endpointとして重要であるrelapse free survivalをprimary endpointとして設定した。

【再発の定義】

　以下の通り再発を定義する。（測定可能病変、測定不能病変の定義についてはRECIST に準ずる[^10^](#_ENREF_10)。）

1. 術後フォローアップのCT検査において測定可能な下記のいずれかの新規病変を認めた時。
2. 腫瘍病変 (最大長径 10mm 以上)　② リンパ節病変 (最大短径 15mm以上)
3. 以下に述べる測定不能病変を有し、かつ腫瘍マーカーが施設における正常上限を超えている時。

測定不能病変：小病変 (長径が10mm未満の腫瘍病変または短径が10mm以上15mm未満であるリンパ節病変、および真の測定不能病変を含む、測定可能病変以外のすべての病変。真の測定不能病変とみなされる病変には次のものがある。軟膜髄膜病変、腹水、胸水または心嚢水、炎症性乳がん、皮膚や肺のリンパ管症、視触診では認識できるが再現性のある画像検査法では測定可能でない腹部腫瘤や腹部臓器の腫大。

無再発生存期間とは術後から上記の再発の定義を満たした日あるいは死亡までの日数と定義

する。

## **副次的評価項目**

　① 全生存率、② 術後再発部位、③術後有害事象発生割合とした。

【設定根拠】

　①は試験治療の治療成績の真の評価の指標として、②は試験治療群における再発形式を知る指標として重要であるために設定した。③は試験の安全性を確保する上で重要と考え設定した。

# **データの集計**

## **記録用紙（Case report form: CRF）**

本試験で用いるCRFは以下のとおり

(1) 症例登録書

(2) 登録適格性確認票

(3) 治療前報告

(4) 手術所見記録

(5) 術後記録 1、2

(6) 病理所見記録

(7) 経過報告書

(8) 治療終了報告

## **記録の保管**

試験の実施等に関わる(1)-(3), (5)の文書は研究事務局で保管する。保管は施錠できるロッカーで行うこととし、鍵は研究代表者が保管する。(4)は各参加施設で管理を行う。保管期間は、研究成果の発表後5年を経過した日までとする。

(1)　申請書類の控え

(2)　倫理委員会からの通知文書

(3)　各種申請書・報告書の控え

(4)　同意書，被験者の同意に関する記録

(5)　CRF

# **統計解析**

## **データの取り扱い**

　実施計画書から逸脱した方法によって得られたデータは、試験終了までに医学専門家等と確認するとともに、計画段階で規定されていない問題への対処について協議決定する。

また計画書に規定された観察・検査項目のデータについて、時期別の集計を行う際には、許容範囲から外れた日もしくは時間に実施した検査項目のデータは欠損扱いとする。欠損値に対し、推定値または計算値などによる補完は行わない。

## **有効性解析**

解析手法の記述に先立ち、解析対象集団を定義する。

「5.1. 登録の手順」に従って登録された患者のうち、重複登録や誤登録を除いた集団を「全登録患者」とする。全登録患者から「不適格患者」を除く集団を「全適格患者」とする。解析対象症例は「全適格患者」と定義する。

本試験では全適格症例において得られた無再発生存期間の90%信頼区間の下限が閾値である0.86年を上回っていた時の本試験治療が有効であると判定する。

## **安全性解析**

有害事象/有害反応の評価には「有害事象共通用語規準v4.02（MedDRA 12.0/MedDRA-J 12.1 対応）日本語訳JCOG 版」（Common Terminology Criteria for Adverse Events v4.0（CTCAE v4.0）の日本語訳）、Clavien-Dindo 分類を用いる。

有害事象のgrading に際しては、それぞれGrade 0～4 の定義内容にもっとも近いものにgrading する。また、Grade に具体的な処置が記載されている場合は、その臨床的な必要性からgrading する。例えば、患者の胸水が増えており、酸素吸入や胸腔ドレナージが適応となる状況にも関わらずそれを患者が拒否した場合などがある。こうした場合には、実際に治療が行われたかどうか（what was actually done）ではなく、何がなされるべきであったか（what should be done）という医学的判断に基づいてgrading を行う。

「8.2.術中・術後の評価項目」で規定された有害事象項目については、該当する記録用紙（治療経過記録用紙）にGrade とそのGrade の発現日を記載する。それ以外の有害事象についてはGrade 3 以上が観察された場合のみ治療経過記録用紙の自由記入欄に有害事象項目とGrade およびそのGrade の発現日を記載する。

# **試験実施期間**

症例登録期間：　承認後　から　2019年　3月　31日（登録締切　2019　年　3月　31日）

症例追跡期間：　承認後　から　2022年　3月　31日

# **倫理的事項**

## **指針及びヘルシンキ宣言への対応**

本試験は人を対象とする医学系研究に関する倫理指針（2014年12月22日制定　文部科学省、厚生労働省）およびヘルシンキ宣言を遵守して実施する。

## **倫理委員会の承認**

本研究は大阪市立大学医学部附属病院の審査委員会の承認を得て実施する。

## **同意・説明文書及び被験者への情報提供**

各施設の審査委員会等で承認の得られた説明文書・同意文書を患者さんに渡し、文書および口頭による十分な説明を行い、患者さんの自由意思による同意を文書で得る。

患者さんの同意に影響を及ぼすと考えられる有効性や安全性等の情報が得られたときや、患者さんの同意に影響を及ぼすような実施計画等の変更が行われるときは、速やかに患者さんに情報提供し、試験等に参加するか否かについて患者さんの意思を予め確認するとともに、事前に各施設の審査委員会等の承認を得て説明文書・同意文書等の改訂を行い、患者さんの再同意を得る。

説明文書には以下の項目を含めるものとする。

1. はじめに：自主臨床試験について

2. あなたの病状

3. この臨床試験の目的

4. この試験の方法

5. この試験の予定参加期間

6. 予測される利益およびリスク

7. この試験に参加されない場合の治療法とその内容

8. 治療にかかる費用

9. この試験を中止させていただく場合があること

10. この試験への参加に同意された場合に守っていただくこと

11. この試験中に、あなたの健康に被害が生じた場合について

12. 研究から生じる知的財産権と利益相反について

13. 　この試験への参加は、あなたの自由意思によること（機器の試験においては試験の参加を取りやめる場合の機器の取扱いに関する事項を含む）

14. この試験に関する情報は、随時ご連絡します

15. この試験結果が公表される場合でも、プライバシーは守られること

16. 　この試験に参加された場合、あなたのカルテなどが試験中あるいは試験終了後に調査されることがあること

17. 資料（試料を含む）の保存、研究終了後の利用又は廃棄方法

18. 相談窓口

## **人権への配慮（個人情報の保護）**

実施に際しては、被験者の人権、福祉及び安全性を最大限に確保する。試験責任医師または分担医師は、本試験の実施にあたっては倫理的な配慮を慎重にし、試験内容について十分説明した上で、患者本人の同意を文書により得る。試験に参加するか否かは、被験者本人の自由意思により決定され、同意後であっても、被験者本人の意思によりいつでも中止が可能である。また、参加中止に伴う不利益は受けない。本試験で知り得た情報は、個人が同定できる形ではいかなる状況においても公表せず、かつ厳重な管理下で保管される。また、本人が希望すれば、本人の情報は本人にのみ文書にて報告する。

## **安全性・不利益への配慮**

　試験治療に伴い有害事象の増加する可能性は低いと考えるが、これらの情報はsecondary endpointsとして情報収集される予定で、有害事象が予期された範囲かどうかについては年2回、研究事務局および効果・安全性評価委員会にてモニタリングを行う。

　また本試験参加中に被験者に何らかの有害事象が発現した場合、担当医師は速やかに必要な対応（検査、治療、試験中止等）を行い、被験者の安全の確保に努める。

## **相談等の対応**

研究対象者又はその代諾者等及びその関係者からの相談、問合せ、苦情等に適切かつ迅速に対応する。

# **試験の費用負担**

## **試験資金および利益相反**

本試験に必要な費用は捺印細胞診の際のパパニコロー染色に要する費用であり、これらは教室研究費、奨励寄付金、文部科学省科学研究費補助金など各種研究に対する助成金により実施する。

## **患者の費用負担**

本試験における試験治療は保険診療の範囲内で実施される。17-1記載の捺印細胞診に関する費用を除き保険適応外の薬剤投与および検査実施は行わない。また、被験者の通院回数、検査実施頻度も通常の診療と同程度であり、試験参加による被験者の負担増加はないと考えられる。

## **健康被害の補償および保険への加入**

本試験に起因して健康被害が生じた場合も、通常の保険診療と同様に患者の健康保険を用いて治療を行う。その際の医療費の自己負担分の支払い、休業補償、差額ベッド料金の補填などの補償については、この研究に関わる組織・施設、および医師等の個人のいずれも被験者に対して補償は行わない。ただし、最善の治療が病院全体で行われるように、担当医師が迅速かつ適切な対応を行う。

# **記録の保存**

　試験責任医師は、試験等の実施に係わる文書（申請書類の控え、研究機関の長からの通知文書、各種申請書・報告書の控、被験者識別コードリスト、同意書、症例報告書等の控、その他データの信頼性を保証するのに必要な書類または記録など）を癌分子病態制御学講座医局にて試験終了5年間保存する。

# **試験計画の登録および試験結果の公表**

　試験実施期間終了後、データ固定後にすべてのエンドポイントに対する解析を行う。研究事務局は可及的速やかにその成果をまとめ、しかるべき英文誌および学会に公表する。最終解析結果の報告は試験実施期間終了後に行う。また、結果の最終の公表を行ったときは、遅滞なく病院長へ報告する。

# **試験組織**

## **研究代表者**

大平雅一

大阪市立大学大学院医学研究科　腫瘍外科学講座

E-mail： masaichi@med.osaka-cu.ac.jp

〒545-8585  大阪市阿倍野区旭町1-4-3

06-6645-3838（代）FAX 06-6646-6450

## **実施施設・試験責任医師**

大平雅一

大阪市立大学大学院医学研究科　腫瘍外科学、癌分子病態制御学

E-mail： masaichi@med.osaka-cu.ac.jp

〒545-8585  大阪市阿倍野区旭町1-4-3

06-6645-3838（代）FAX 06-6646-6450

久保尚士

大阪市立総合医療センター　消化器外科

E-mail： k-naoshi@med.osaka-cu.ac.jp

06-6929-1221（代）FAX 06-6929-1090

## **試験事務局**

三木友一朗

大阪市立大学大学院医学研究科　腫瘍外科学、癌分子病態制御学

E-mail： y_miki@ med.osaka-cu.ac.jp

〒545-8585  大阪市阿倍野区旭町1-4-3

06-6645-3838（代）FAX 06-6646-6450

八代正和

大阪市立大学大学院医学研究科　腫瘍外科学、癌分子病態制御学

E-mail： m9312510@med.osaka-cu.ac.jp

〒545-8585  大阪市阿倍野区旭町1-4-3

06-6645-3838（代）FAX 06-6646-6450

## **効果安全性評価委員会**

澤田鉄二

大阪掖済会病院　院長

[t.sawada@osaka-ekisaikai.jp](mailto:t.sawada@osaka-ekisaikai.jp)

松岡翼

なにわ生野病院　外科部長

tsubasam1965@yahoo.co.jp

## **モニタリング担当者（モニター）**

永原央

大阪市立大学大学院医学研究科　腫瘍外科学、癌分子病態制御学

E-mail： hisashi@ med.osaka-cu.ac.jp

〒545-8585  大阪市阿倍野区旭町1-4-3

06-6645-3838（代）FAX 06-6646-6450

## **生物統計家**

福井充

大阪市立大学大学院医学研究科　都市医学講座

E-mail： fukui@ med.osaka-cu.ac.jp

〒545-8585  大阪市阿倍野区旭町1-4-3

06-6645-3770

# **実施計画書からの逸脱または変更**

実施計画書や説明文書・同意文書の改訂を行う場合は予め臨床試験・治験審査委員会の承認を必要とする。ただし、被験者の緊急の危険を回避するためのものであるなど医療上やむを得ないものに関する変更である場合には、この限りではない。

試験責任医師または試験分担医師は、試験実施計画書からの重大な逸脱があった場合は、逸脱事項をその理由とともに記録する。

試験責任医師は、試験薬の品質、有効性及び安全性に関する事項、その他試験を訂正に行うために重要な情報を知ったときに、必要に応じて実施計画書を改訂する。有効性試験実施計画書からの重大な逸脱があった場合は、逸脱事項をその理由とともに記録する。

# **モニタリング／データの品質管理**

本試験では、研究の科学的・倫理的な質の向上と教育を目的として施設訪問モニタリングを行うことがある。モニターは、研究責任者が要求する事項について確認することにより、臨床研究が適切に実施されていること、必要な事項が正確に記録されていること及びデータの信頼性が十分に保たれていることを保証する。

# **監査**

本試験では、研究の科学的・倫理的な質の向上と教育を目的として施設訪問監査を行うことがある。施設の選定は、別途定める監査の実施に関する手順書に記載の方法で決定される。

# **参考文献**

**1.** 厚生統計協会. *国民衛生の動向.* Vol 42.

**2.** Sakuramoto S, Sasako M, Yamaguchi T, et al. Adjuvant chemotherapy for gastric cancer with S-1, an oral fluoropyrimidine. *N Engl J Med.* Nov 1 2007;357(18):1810-1820.

**3.** Noh SH, Park SR, Yang HK, et al. Adjuvant capecitabine plus oxaliplatin for gastric cancer after D2 gastrectomy (CLASSIC): 5-year follow-up of an open-label, randomised phase 3 trial. *Lancet Oncol.* Nov 2014;15(12):1389-1396.

**4.** Sasako M, Sakuramoto S, Katai H, et al. Five-year outcomes of a randomized phase III trial comparing adjuvant chemotherapy with S-1 versus surgery alone in stage II or III gastric cancer. *J Clin Oncol.* Nov 20 2011;29(33):4387-4393.

**5.** Bando E, Yonemura Y, Takeshita Y, et al. Intraoperative lavage for cytological examination in 1,297 patients with gastric carcinoma. *American journal of surgery.* Sep 1999;178(3):256-262.

**6.** Kodera Y, Imano M, Yoshikawa T, et al. A randomized phase II trial to test the efficacy of intra-peritoneal paclitaxel for gastric cancer with high risk for the peritoneal metastasis (INPACT trial). *Jpn J Clin Oncol.* Feb 2011;41(2):283-286.

**7.** Kuramoto M, Shimada S, Ikeshima S, et al. Extensive intraoperative peritoneal lavage as a standard prophylactic strategy for peritoneal recurrence in patients with gastric carcinoma. *Ann Surg.* Aug 2009;250(2):242-246.

**8.** Masuda T, Kuramoto M, Shimada S, et al. The effect of extensive intraoperative peritoneal lavage therapy (EIPL) on stage III B + C and cytology-positive gastric cancer patients. *International journal of clinical oncology.* Apr 2016;21(2):289-294.

**9.** Fujitani K, Yang HK, Kurokawa Y, et al. Randomized controlled trial comparing gastrectomy plus chemotherapy with chemotherapy alone in advanced gastric cancer with a single non-curable factor: Japan Clinical Oncology Group Study JCOG 0705 and Korea Gastric Cancer Association Study KGCA01. *Jpn J Clin Oncol.* Jul 2008;38(7):504-506.

**10.** Eisenhauer EA, Therasse P, Bogaerts J, et al. New response evaluation criteria in solid tumours: revised RECIST guideline (version 1.1). *European journal of cancer (Oxford, England : 1990).* Jan 2009;45(2):228-247.
